# Exploring the role of microbiome in cystic fibrosis clinical outcomes through a mediation analysis

**DOI:** 10.1128/msystems.00196-25

**Published:** 2025-05-28

**Authors:** Seda Sevilay Koldaş, Osman Uğur Sezerman, Emel Timuçin

**Affiliations:** 1 Biostatistics and Bioinformatics, School of Health Science, Acıbadem Mehmet Ali Aydınlar University 162328, , Istanbul, Turkey; 2 Biostatistics and Bioinformatics, School of Health Science, Acıbadem Mehmet Ali Aydınlar University 162328, Istanbul, Turkey; 3 Molecular Biology and Genetics, Faculty of Science, Gebze Technical University, Kocaeli, Turkey; Georgia Institute of Technology, Atlanta, Georgia, USA

**Keywords:** mediation analysis, structural equation modeling, cystic fibrosis, longitudinal data, linear mixed effects model, high-dimensional

## Abstract

Human microbiome plays a crucial role in host health and disease by mediating the impact of environmental factors on clinical outcomes. Mediation analysis is a valuable tool for dissecting these complex relationships. However, existing approaches are primarily designed for cross-sectional studies. Modern clinical research increasingly utilizes long follow-up periods, leading to complex data structures, particularly in metagenomic studies. To address this limitation, we introduce a novel mediation framework based on structural equation modeling that leverages linear mixed-effects models using penalized quasi-likelihood estimation with a debiased lasso. We applied this framework to a 16S rRNA sputum microbiome data set collected from patients with cystic fibrosis over 10 years to investigate the mediating role of the microbiome in the relationship between clinical states, disease aggressiveness phenotypes, and lung function. We identified richness as a key mediator of lung function. Specifically, *Streptococcus* was found to be significantly associated with mediating the decline in lung function on treatment compared to exacerbation, while *Gemella* was associated with the decline in lung function on recovery. This approach offers a powerful new tool for understanding the complex interplay between microbiome and clinical outcomes in longitudinal studies, facilitating targeted microbiome-based interventions.

**IMPORTANCE:**

Understanding the mechanisms by which the microbiome influences clinical outcomes is paramount for realizing the full potential of microbiome-based medicine, including diagnostics and therapeutics. Identifying specific microbial mediators not only reveals potential targets for novel therapies and drug repurposing but also offers a more precise approach to patient stratification and personalized interventions. While traditional mediation analyses are ill-equipped to address the complexities of longitudinal metagenomic data, our framework directly addresses this gap, enabling robust investigation of these increasingly common study designs. By applying this framework to a decade-long cystic fibrosis study, we have begun to unravel the intricate relationships between the sputum microbiome and lung function decline across different clinical states, yielding insights that were previously unknown.

## INTRODUCTION

**Fig 1:**
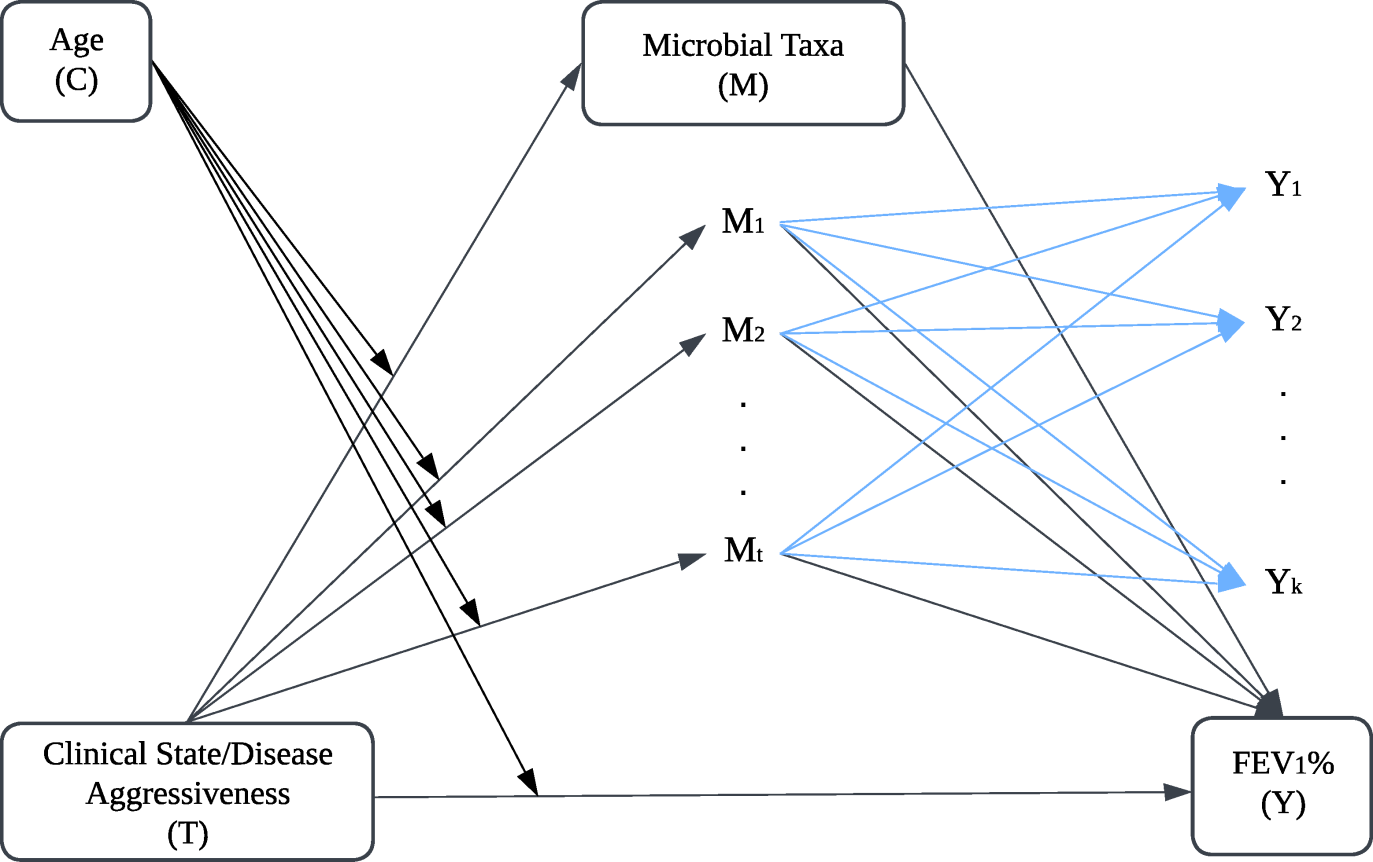
Directed acyclic path diagram of SEM-based mediation model. The diagram illustrates a high-dimensional mediation model with repeated measures. It shows how sputum microbial taxa (M) mediate the relationship between clinical state or disease aggressiveness (T) and FEV_1_% (Y), with age (C) as a potential covariate.

Microbiome constitutes a dynamic community of microorganisms interacting intricately with hosts and environments, significantly influencing health outcomes, including treatment responses (1–3). Prior research has established links among the environment, microbiome, and health (4–8). Mediation analysis is a statistical technique used to examine how an independent variable (*T*) affects a dependent variable (*Y*) directly or indirectly through a mediator (*M*), aiming to explain the underlying mechanism or pathway of this relationship (Fig. 1). This approach helps understand microbiome influences on clinical outcomes by elucidating how environmental or treatment-related changes affect microbiome composition (9), enabling novel microbiome-based interventions (10, 11). As clinical research advances, studies increasingly use long follow-up periods, resulting in complex data structures. To enhance mediation analysis and capture random effects within subjects inherent to this type of data, linear mixed-effects models are effective tools for handling repeated measures and clustered data by incorporating both fixed and random effects, accommodating inherent correlation structures in complex data sets (12). However, estimating and drawing inferences about fixed effects in linear mixed-effects models can be challenging in high-dimensional settings. Recently, Li et al. proposed a penalized quasi-likelihood approach for fixed effects estimation in high-dimensional linear mixed-effects models. Their approach applies broadly to repeated measures, particularly with large or heterogeneous clusters, and avoids strong distributional assumptions for random effects and errors (13). Cui et al. addressed high-dimensionality in a longitudinal study using sure independence screening for variable selection, modeled mediation effects with linear mixed-effects, and generalized estimating equations. They found that the mixed-effects model yielded more accurate indirect effect estimates (14).

In metagenomic studies, the dimensions of microbial features can be exceptionally large, possibly much larger than the sample size. Although various statistical methods exist for high-dimensional microbiome mediation analysis (8, 15–19), most are designed for cross-sectional data. Therefore, there is a need for new mediation analysis methods that can handle high-dimensional data generated by repeated measures and longitudinal clinical cohorts (14). Structural equation modeling (SEM) addresses this need by explicitly modeling mediation and managing repeated measures by linking regression models through conceptual path diagrams. This is particularly advantageous for analyzing dynamic relationships between observed and latent variables (20, 21). SEM can also be adapted to high-dimensional data. Therefore, we propose an approach for estimating and testing mediators that utilizes a SEM-based mediation framework using the product-of-coefficient method (22).

Etiology of cystic fibrosis (CF), including lung function and disease progression, is linked to microbiome changes (23). The respiratory microbiome in CF evolves with age and is shaped by baseline disease state and progression rate (24). While stable lung function is associated with a more diverse microbiome, it remains lower than that in healthy individuals, indicating an altered microbial ecology in CF. Conversely, declining lung function correlates with reduced diversity and increased dominance of pathogens, especially *Pseudomonas aeruginosa* (25, 26). This shift, partly driven by recurrent antibiotic use, is associated with increased disease severity (26–30). Importantly, these alterations are not only a consequence of CF but also contribute to its progression by sustaining inflammation and infection (30, 31).

In light of these research, we aim to explore the mechanisms by which the microbiome influences the relationship between clinical state and lung health as measured by forced expiratory volume in one second (FEV_1_%) (Fig. 1), as well as the relationship between the disease aggressiveness phenotype and FEV_1_%. Our case study involves sputum microbiome data collected from CF patients over 10 years and originally published by Widder et al. and Carmody et al. (32, 33). Given the inherent challenges of microbiome data, such as sparsity, over-dispersion, multicollinearity, and high dimensionality (34, 35), we first apply a log-transformation of proportions to address sparsity and compositionality (36, 37). To tackle multicollinearity arising from high-dimensional data, we employ the penalized quasi-likelihood approach based on the debiased lasso technique, complemented by a bias-corrected bootstrapping method to evaluate the mediation effects in repeated measurement/longitudinal microbiome data (13, 38).

We also build a univariate SEM-based mediation framework using linear mixed-effects models for community-level microbiome metrics, such as alpha diversity.

## RESULTS

### Description of the study design

We analyzed 631 sputum samples from 111 CF patients. These samples had been collected as part of standard medical care and subsequently obtained from the University of Michigan Health System’s clinical microbiology laboratory. The patients and sample characteristics are summarized in Table 1. Samples representing various clinical states were collected unevenly across participants and time points with highly variable sampling intervals (days to years), and clinical states were not always consecutive. Clinical state at the time of sample collection was defined as exacerbation (exacerbation before antibiotic administration); treatment (antibiotic treatment for exacerbation); and recovery (recovery from exacerbation within 21 days after cessation of antibiotics) (32, 39).

**TABLE 1:** Patient and sample characteristics

Variable	Values
No. of patients	111
Patient age, mean (range)	26 (6–54)
Patient FEV_1_%, mean (range)	58 (16–125)
No. of samples	631
No. of samples per patient, mean (range)	5 (1–24)
Disease aggressiveness, count (%)	
Mild	178 (51)
Moderate/severe	172 (49)
Clinical state, count (%)	
Baseline	268 (43)
Exacerbation	127 (20)
Treatment	113 (18)
Recovery	123 (19)

To evaluate the impact of the disease aggressiveness phenotype, we analyzed a longitudinal cohort of 24 patients (350 samples total), each providing at least 10 sputum samples collected over a minimum of 5 years. Phenotypes, which are categorized as mild, moderate, or severe, were determined based on the rate of FEV_1_% decline relative to age (40). In the mild phenotype, the clinical state proportions were 57% at baseline, 17% exacerbation, 11% treatment, and 15% recovery; in the moderate/severe phenotype, they were 32%, 19%, 29%, and 20%, respectively. Patient age (mean 27, range 11–54 years), FEV_1_% (mean 56%, range 18%–101%), and sample characteristics are available in Table S1.

We excluded operational taxonomic units (OTUs) identified as Archaea and Eukarya from the analysis. Subsequently, we aggregated the remaining OTUs at the genus level, which yielded 196 bacterial genera. For community-level analysis, we utilized these 196 bacterial genera. We retained genera with over 3 counts in at least 5% of the samples, resulting in 47 genera for microbial taxa mediation analysis. Furthermore, to accommodate the longitudinal design of the mediation framework for evaluating the impact of clinical states, we excluded patients with a single sample, resulting in a final cohort size of 89 patients with 599 samples.

### Relationships of microbiome, FEV_1_%, and clinical outcomes

**Fig 2:**
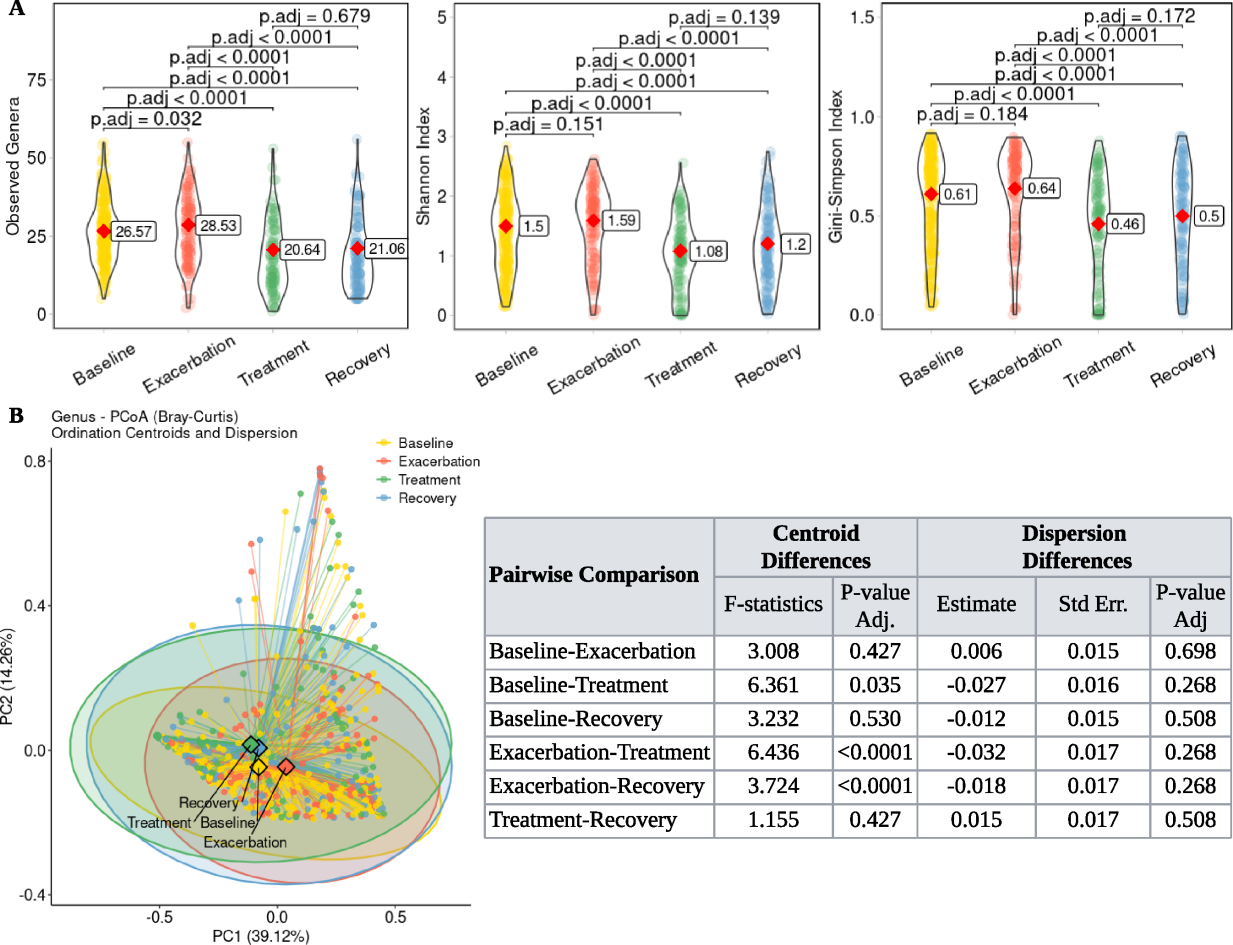
Changes in sputum microbiome diversity and composition across clinical states. (**A**) Alpha diversity metrics, including observed, Shannon, and Simpson indices, were calculated at genus level. Violin plots show the distribution of each index within clinical states, with embedded red diamonds indicating estimated marginal means, derived from linear mixed-effects model (LMM) accounting for within-subject correlations. The corresponding estimated marginal mean value for each clinical state is displayed in the box on the right. Pairwise statistical comparisons were performed on estimated marginal means derived from LMM. *P*-values are shown at the top of the figure. (**B**) Principal coordinate analysis (PCoA) was performed on genus-level compositions using Bray-Curtis dissimilarity. Samples are plotted and colored by clinical states. The ellipses illustrate the dispersion of samples within each group, reflecting the variability in genus composition. The lines connecting each individual sample point to its group centroid help visualize the dispersion. The percentage of variance explained by each axis is shown in parentheses. The diamond symbols represent the centroid of each group, indicating the average community composition. Differences in group centroids (average community composition) were tested using pairwise PERMANOVA. Differences in dispersion (variability) around these centroids were assessed using LMM accounting for within-subject correlations. *P*-values are adjusted using the Benjamini-Hochberg correction, corresponding scores are tabled on the right side of the plot.

**Fig 3:**
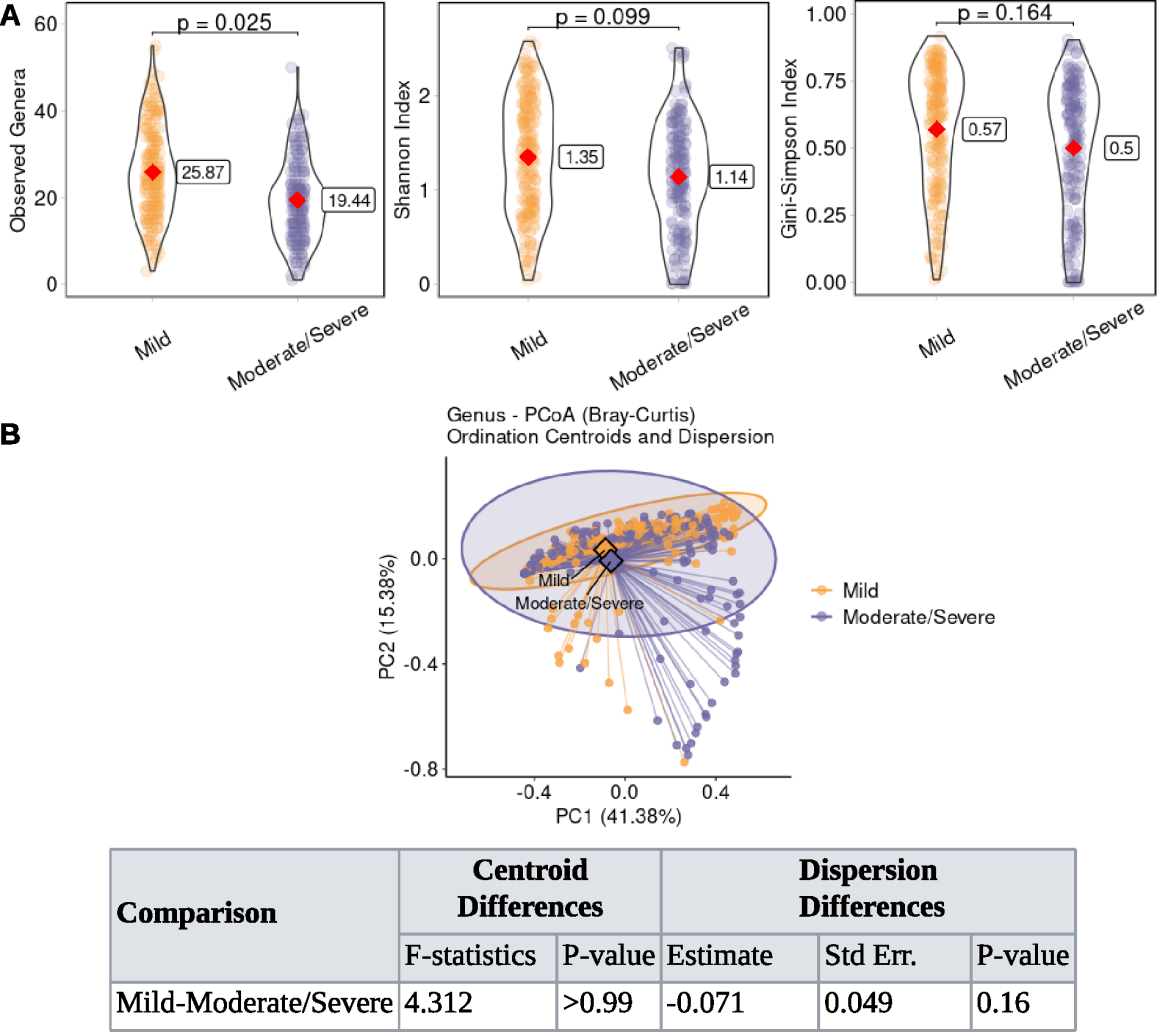
Changes in sputum microbiome diversity and composition across disease aggressiveness. (**A**) Alpha diversity metrics, including observed, Shannon, and Simpson indices, were calculated at genus level. Violin plots show the distribution of each index within disease aggressiveness phenotypes, with embedded red diamond indicating estimated marginal means, derived from linear mixed-effects model (LMM) accounting for within-subject correlations. The corresponding estimated marginal mean value for each aggressiveness phenotype is displayed in the box on the right. Pairwise statistical comparisons were performed on estimated marginal means derived from LMM. *P*-values are shown at the top of the figure. (**B**) Principal coordinate analysis (PCoA) was performed on genus-level compositions using Bray-Curtis dissimilarity. Samples are plotted and colored by disease aggressiveness phenotype. The ellipses illustrate the dispersion of samples within each group, reflecting the variability in genus composition. The lines connecting each individual sample point to its group centroid help visualize the dispersion. The percentage of variance explained by each axis is shown in parentheses. The diamond symbols represent the centroid of each group, indicating the average community composition. Differences in group centroids (average community composition) were tested using PERMANOVA. Differences in dispersion (variability) around these centroids were assessed using LMM accounting for within-subject correlations. Corresponding scores are tabled at the bottom of the plot.

Alpha diversity, a key measure of within-sample microbial diversity, helps characterize community structure and function. To assess its impact on lung function, we used observed, Shannon, and Simpson indices. The observed index quantifies richness, reflecting the total number of unique genera; Shannon incorporates both richness and evenness, emphasizing the balance of genera abundances; Simpson focuses on dominance, reflecting the likelihood that two individuals belong to the same genus. These complementary indices provide a comprehensive view of community structure, encompassing richness, evenness, and dominance (41, 42). Analysis of the alpha diversity revealed no significant differences between the treatment and recovery phases for any index. However, observed richness was significantly higher in exacerbation than baseline (*P* = 0.032), while Shannon and Simpson indices did not differ (Fig. 2A). By disease aggressiveness phenotype, observed richness was significantly greater in the mild phenotype than in moderate/severe (*P* = 0.025), but Shannon and Simpson indices showed no significant differences (Fig. 3A).

Principal coordinate analysis (PCoA) using Bray-Curtis distance visualized variance in genus-level community composition across samples, reflecting differences in microbial community structure. This ordination facilitated the visual assessment of group distinctions, and PERMANOVA and dispersion tests were used to evaluate statistical significance. The first two principal coordinates explained 53.38% of the total variability in clinical states. PERMANOVA revealed no significant differences between exacerbation/recovery and baseline or between treatment and recovery. However, significant differences were found between the treatment and baseline (*P* = 0.035), treatment and exacerbation (*P* < 0.0001), and exacerbation and recovery (*P* < 0.0001). Microbial communities within each clinical state showed similar dispersion, with no state being more variable than the others (Fig. 2B). Regarding disease aggressiveness, the first two coordinates explained 56.76% of the variability. No significant compositional or dispersion differences were observed between the mild and moderate/severe phenotypes (Fig. 3B).

FEV_1_% is a key tool for diagnosing and monitoring respiratory health. We compared FEV_1_% across clinical states and disease aggressiveness phenotypes using linear mixed-effects models to account for repeated measures. Pairwise comparisons of estimated marginal means showed that baseline FEV_1_% was significantly higher than treatment (14%, SE = 0.03, *P* < 0.0001) and recovery (16%, SE = 0.03, *P* < 0.0001), but not exacerbations (5%, SE = 0.02, *P* = 0.06). Exacerbation FEV_1_% was significantly higher than treatment (9%, SE = 0.03, *P* = 0.004) and recovery (11%, SE = 0.03, *P* = 0.0003), while treatment and recovery did not differ (SE = 0.03, *P* = 0.45). Regarding disease aggressiveness, FEV_1_% was 62% higher in the mild phenotype than in the moderate/severe phenotype (SE = 0.16, *P* = 0.0001).

### Role of the sputum microbiome as a mediator

We next investigated whether clinical states and disease aggressiveness phenotypes affect sputum microbial taxa or diversity in CF patients and whether these changes affect FEV_1_%. To account for repeated measures, our proposed approach used linear mixed-effects models with random intercepts to partially capture individual variability. However, the analysis did not explicitly incorporate time as a continuous covariate or account for uneven temporal intervals between sampling points. Therefore, while random effects were controlled for patient-specific variability, they did not fully address the potential effects of disease progression over time.

#### Microbial taxa mediation

Compared to the baseline, FEV_1_% significantly decreased by 5.048 units in exacerbation, 5.908 in treatment, and 6.403 in recovery. After accounting for microbial taxa as potential mediators, these reductions were slightly attenuated to 4.801, 4.601, and 4.322 units, respectively, though, no microbial taxa significantly mediated these changes (Table 2; Fig. S1A through C).

**TABLE 2:** The total and direct effects of clinical states and disease aggressiveness mediated by sputum microbial taxa on FEV_1_%

Parameter	Clinical outcomes		Estimate	CI-lower	CI-upper	*P*-value
Clinical states	Baseline-exacerbation	Total effect	−5.048	−7.363	−2.747	< 0.001
		Direct effect	−4.801	−7.018	−2.560	< 0.001
	Baseline-treatment	Total effect	−5.908	−9.348	−2.614	0.001
		Direct effect	−4.601	−7.542	−1.603	0.003
	Baseline-recovery	Total effect	−6.403	−10.488	−2.617	< 0.001
		Direct effect	−4.322	−7.752	−0.912	0.009
	Exacerbation-treatment	Total effect	−3.323	−8.809	1.339	0.193
		Direct effect	0.173	−3.807	3.804	0.905
	Exacerbation-recovery	Total effect	−4.096	−9.418	0.731	0.097
		Direct effect	0.120	−3.307	3.438	0.921
	Treatment-recovery	Total effect	−1.469	−5.610	2.748	0.482
		Direct effect	−0.507	−3.274	2.342	0.710
Disease aggressiveness	Mild-moderate/severe	Total effect	−21.061	−30.587	−12.464	< 0.001
		Direct effect	−20.366	−30.262	−11.774	< 0.001

**Fig 4:**
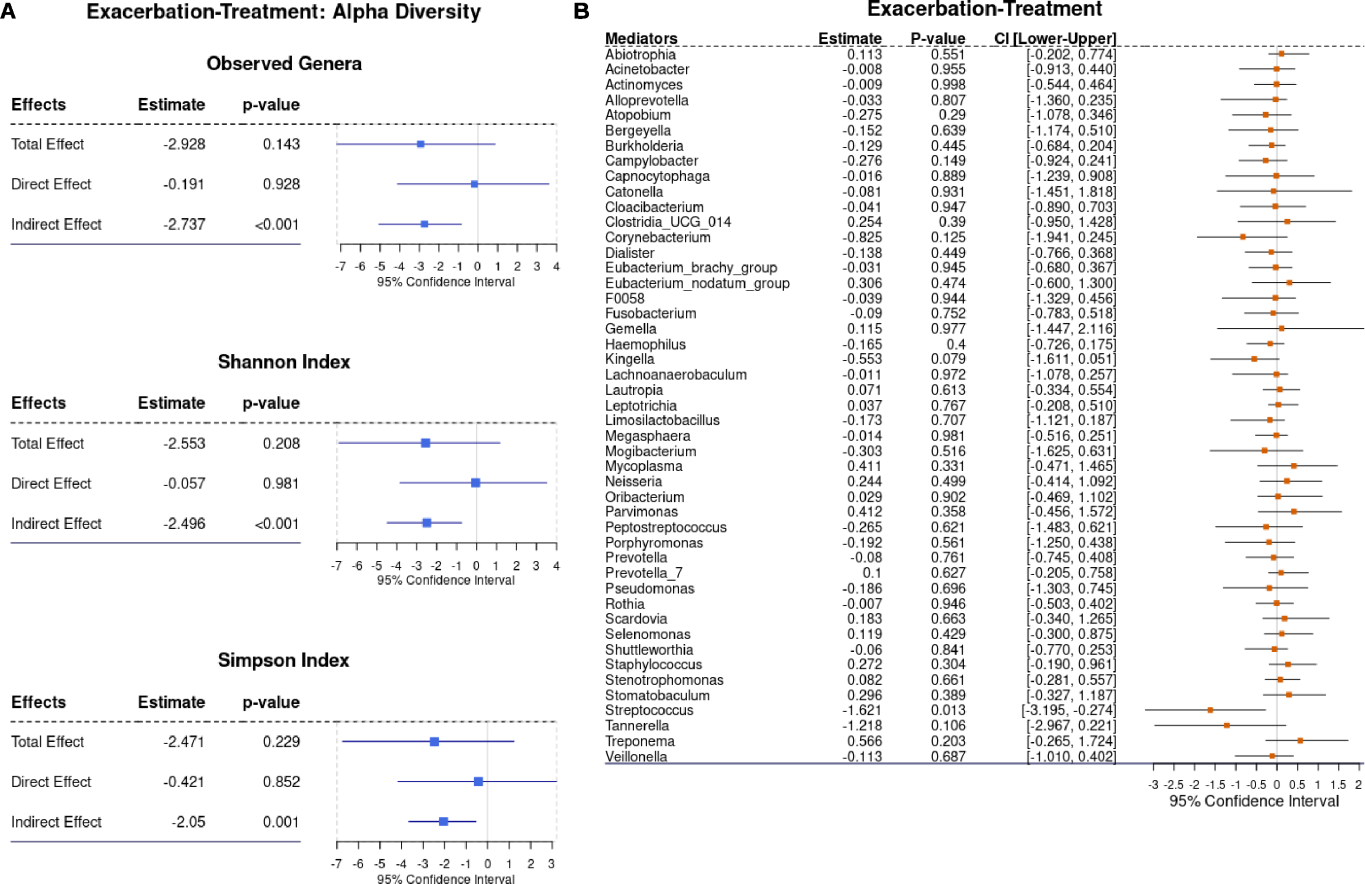
Results for alpha diversity and bacterial genera in the comparison of exacerbation vs treatment. (**A**) The plots present the results of each of the alpha diversity metrics (Observed, Shannon, and Simpson) that were evaluated as a potential mediator in the causal pathway between clinical states and FEV_1_%. Alpha diversity metrics were calculated at the genus level. (**B**) The forest plot illustrates the influence of the sputum microbiome on the relationship between clinical states and FEV_1_%. The sputum microbial taxa are presented at the genus level. The confidence intervals and *P*-values were obtained using a nonparametric bootstrap approach with 3,000 resamples. “Estimate” represents the mean of all bootstraps resamples. Results are presented with a 95% confidence interval.

Treatment was associated with a non-significant 3.323-unit decrease in FEV_1_% vs exacerbation, which shifted to a non-significant 0.173-unit increase after accounting for microbial taxa (Table 2). Notably, only *Streptococcus* significantly contributed to a 1.621-unit FEV_1_% decrease (*P* = 0.013; 95% CI: −3.195, −0.274) (Fig. 4B).

Recovery showed a non-significant 4.096-unit FEV_1_% decrease relative to exacerbation, attenuating to a 0.120-unit increase after accounting for microbial taxa (Table 2). Only *Gemella* significantly contributed to a 0.94-unit FEV_1_% decrease (*P* = 0.049; 95% CI: −2.166, 0.013) (Fig. S1D).

Compared to the treatment, recovery was associated with a non-significant 1.469-unit FEV_1_% decrease, shifting to a 0.507-unit decrease after adjustment for microbial taxa (Table 2). *Gemella* was the only significant contributor, associated with a 0.616-unit FEV_1_% decrease (*P* = 0.038; 95% CI: −1.580, 0.033) (Fig. S1E).

Finally, the moderate/severe phenotype was associated with a 21.061-unit FEV_1_% decrease compared to the mild phenotype, slightly reduced to 20.366 units after adjusting for microbial taxa, and none of the microbial taxa demonstrated significant mediation (Table 2; Fig. S1F).

#### Community-level mediation

Exacerbation, treatment, and recovery were each associated with significant FEV_1_% reductions compared to baseline. While these reductions were primarily due to the direct effects of clinical states, the observed, Shannon, and Simpson indices mediated the effects in treatment and recovery, but not in exacerbation (Fig. S2A through C). Treatment and recovery had no significant direct or total effects on FEV_1_% relative to exacerbation, yet all three indices significantly mediated the effects in both comparisons (Fig. 4A; Fig. S2D). Recovery showed no significant direct, total, or mediating effects (including Observed, Shannon, and Simpson indices) on FEV_1_% compared to treatment (Fig. S2E). The moderate/severe phenotype was associated with a significant FEV_1_% reduction compared to the mild phenotype, driven by direct effects. Among the alpha diversity indices, only Observed richness was significantly associated with decreased FEV_1_% (Fig. S2F).

## DISCUSSION

In this study, we extended the high-dimensional linear mixed-effects model proposed by Li et al. (13) into a SEM-based mediation framework to investigate whether the high-dimensional, compositional microbiome mediates the effects of clinical state and disease aggressiveness on FEV_1_%. For taxonomical mediation analysis, our method treats microbiome composition as a unit, estimating exposure effects on outcomes through microbial mediators (43). This method is particularly efficient for repeated measures or longitudinal data. The estimation procedure does not require strong distributional assumptions for random effects or errors (13). We assessed indirect effects and their statistical significance using a non-parametric bootstrap procedure with 3,000 resamples and bias-corrected and accelerated (BCa) confidence intervals. When applying the Benjamini-Hochberg correction, no indirect effects met the *P* ≤ 0.05 threshold, though the true proportion of mediators is usually unknown, as previous studies have indicated (44, 45).

We also performed a single-mediator causal analysis, with alpha diversity as the mediator, using linear mixed-effects models within a SEM framework. In this analysis, we examined each alpha diversity index in separate mediation models to determine its individual contribution to mediating the effect of exposure on the outcome.

Our framework reveals causal mechanisms, not just associations, between the CF lung microbiota and clinical outcomes, extending previous findings from the same data set. Previous studies used generalized estimating equations (GEE) to examine patient-specific factors with microbiome characteristics (33) and identified associations of bacterial community types with lung disease (32). In contrast, our SEM-based mediation analysis investigated whether the microbiome mediates the effects of clinical state and disease aggressiveness phenotype on FEV_1_%. By moving beyond correlations, we provide mechanistic insights into how microbiome changes may drive lung function decline in CF, questions unaddressed by associative approaches. Given FEV_1_’s importance as a clinical outcome, our approach offers more direct, actionable links between microbiome changes and patient health. Unlike GEE, our SEM framework captures indirect effects and complex pathways, laying the groundwork for identifying microbial targets to improve lung function in CF patients.

Our FEV_1_% findings generally align with existing research though some results require nuanced interpretation. FEV_1_% was significantly higher in baseline than treatment and recovery, but not significantly different from exacerbation. Notably, FEV_1_% in exacerbation was higher than treatment and recovery. These patterns suggest better lung function before interventions and that treatment measurements were likely taken very early in the course of acute management, potentially at or near the nadir of lung function decline triggered by the exacerbation. The exacerbation measurements, in contrast, might have been taken slightly earlier in the exacerbation event before the absolute lowest point was reached, or perhaps after a very slight spontaneous improvement, resulting in a transiently higher FEV₁. Prior studies reported that FEV_1_% often declines around exacerbations and does not fully return to baseline during recovery, which may explain our observation. The lack of difference between treatment and recovery FEV_1_% may reflect a common incomplete recovery (46–48).

In our retrospective analysis, observed richness was significantly higher in exacerbation than baseline, treatment, and recovery. While Shannon and Simpson indices in exacerbation did not differ from baseline, they were notably higher than those in treatment and recovery (Fig. 2A), suggesting an influx of rare or transient genera that increased richness without markedly affecting evenness. Thus, while the community maintained a similar evenness, it was significantly more diverse in exacerbation than during or after treatment. This implies that airway microbiome diversity may not return to baseline levels immediately after treatment; both the number of species and overall diversity may remain suppressed during the recovery phase, suggesting a lasting effect of treatment (49, 50). This may reflect residual inflammation, airway remodeling, or incomplete eradication of pathogens like *Pseudomonas* and *Burkholderia* (51). As expected, treatment altered both diversity and composition, and we further found that diversity mediated its effect on FEV_1_%. The main difference between the baseline and exacerbation lay in the number of genera (richness), not the overall community balance (Shannon/Simpson) (Fig. 2A), contrasting with scenarios driven by dominance of a single pathogen. Rather, these changes probably reflect the addition of multiple genera. Regarding disease aggressiveness, observed richness was significantly higher in the mild phenotype than moderate/severe, while Shannon and Simpson indices showed no significant differences (Fig. 3A). This suggests that a milder phenotype might be associated with a greater variety of genera, yet the overall community structure regarding evenness remains similar across phenotypes, implying that the additional genera in the mild phenotype are likely rare community members.

It is important to note that variability in sample collection timing may have influenced our results. For instance, during treatment, samples were taken while patients were receiving IV or oral antibiotics, which indicates a dynamic period of active pathogen reduction and inflammation control. Thus, the exact timing within the treatment and recovery phases could significantly affect the observed microbial communities and inflammatory markers, with early samples showing minimal changes and later samples revealing partial shifts without full resolution.

**Fig 5:**
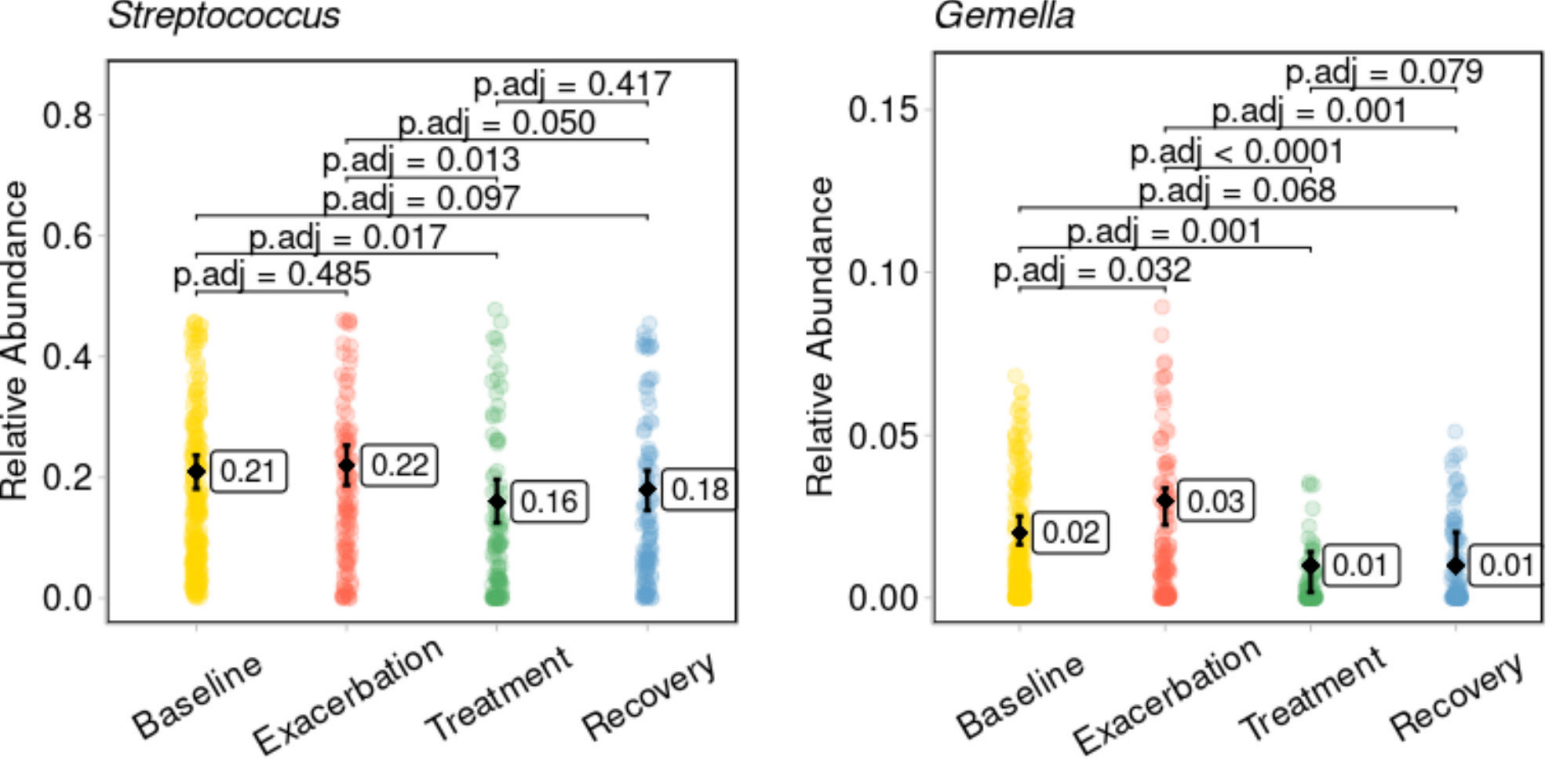
*Streptococcus* and *Gemella* relative abundances across clinical states. *Streptococcus* and *Gemella* relative abundances across clinical states. Each point represents a single sample and is colored according to clinical state. Black diamonds indicate estimated marginal means with 95% confidence intervals, derived from a linear mixed-effects model. The corresponding means are shown in adjacent labels. Pairwise comparisons were performed on the marginal means, and adjusted *P*-values (Benjamini-Hochberg correction) are shown above the plot.

The known CF pathogens such as *Pseudomonas, Streptococcus*, and *Burkholderia* during exacerbations are well-documented, posing a threat via biofilm formation and persistent infection and inflammation (24–26, 51–53). A recent review highlighted *Streptococcus*’s dual role: some streptococci exacerbate inflammation and lung damage, while others correlate with lower disease burden (54). Our mediation analysis identified *Streptococcus* as the only significant mediator among known pathogens, contributing to FEV_1_% decline in the treatment phase compared to exacerbation (Fig. 4B), despite its reduced abundance in the treatment (Fig. 5B). This suggests that even lower *Streptococcus* levels during treatment may impact lung function. The relative abundance of *Gemella* also dropped significantly in treatment and recovery compared to exacerbation, with no difference between treatment and recovery (*P* = 0.079) (Fig. 5B). Notably, *Gemella* consistently mediated FEV_1_% decline in recovery compared to both exacerbation and treatment, suggesting a pathogenic role in preventing return to baseline lung function. Although *Gemella* is part of the healthy oral microbiota, it might trigger pulmonary exacerbations, possibly by remodeling the airway microbiota (51, 55). Both *Streptococcus* and *Gemella* might contribute to lung function decline at different clinical states. Their relative abundances and associations with FEV_1_% underscore their importance as targets for intervention. Effective strategies may include reducing *Streptococcus* during exacerbation and treatment and mitigating *Gemella*’s impact during recovery. Crucially, the broader clinical context and individual patient factors must be considered when assessing FEV_1_% changes in CF.

Disease aggressiveness is a critical predictor of CF progression, treatment guidance, and outcomes. Although retrospectively defined disease aggressiveness limits causal inference, likely introducing potential reverse causation, mechanistic insights from our model reveal actionable therapeutic pathways by highlighting the role of the microbiome. Preserving microbial diversity, targeting early decline, and personalizing treatments based on microbiome profiles may improve outcomes. Konstan et al. reported that aggressive phenotypes, characterized by a faster rate of lung function decline, are also associated with earlier mortality, indicating a broader impact of these phenotypes (40). Similarly, we observed that FEV_1_% in moderate/severe phenotypes was significantly lower than that in mild phenotype. Although microbial taxa did not significantly mediate this difference, observed richness did, consistent with prior findings. Frey et al. also reported reduced microbial diversity in association with lower lung function (25).

We assessed microbial richness using 454 sequencing by counting the observed genera. While advanced at the time, this platform offers limited depth compared to current technologies. Our 1,000-read threshold may have restricted the detection of low-abundance genera, potentially underestimating the overall diversity.

Despite not identifying novel mediators beyond the literature, our SEM framework validates prior findings and reveals new insights into indirect pathways. Most longitudinal microbiome studies rely on basic linear regression or mixed-effects models, which are limited in high-dimensional settings. In contrast, our SEM-based mediation approach enabled a more nuanced analysis.

However, the retrospective nature of our study introduces inherent limitations. Uneven sampling and variable clinical state distributions, such as two patients with mild disease aggressiveness sampled only at baseline, may confound the associations with FEV_1_%. The temporal gaps between samples and variability in exacerbation frequency further complicate the attribution of observed changes to specific clinical states or disease phenotypes. Differences in cumulative antibiotic exposure, especially in more aggressive phenotypes, might have influenced our findings although the antibiotic type, route, and period were not assessed. The lack of key demographic and clinical data (e.g., gender, CFTR genotype) also indicates possible unmeasured confounding.

Furthermore, recent advances in modulator therapies that reduce sputum production and alter its composition may limit its utility for microbial studies. Future research should consider alternative biosampling strategies, such as bronchoalveolar lavage or oropharyngeal swabs, and use standardized, sequential protocols to better capture temporal dynamics.

### Conclusions

Methodologies for longitudinal microbial data remain underdeveloped and require robust frameworks to clarify the biological and temporal relationships among microorganisms. Our study offers a much-needed approach for examining causal relationships in high-dimensional, longitudinal data. While mediation analysis provides stronger evidence than simple association, the observational data we used to test it allows us to provide suggestive evidence and investigate potential mechanisms, but it does not provide definitive proof of causality, unlike randomized controlled trials. Our findings highlight the complex interplay between the sputum microbiome, clinical outcomes, and disease aggressiveness in CF. Identifying key microbial mediators provides potential targets for new or improved therapies. Refining our approach with species-level identification and integrating multi-omics data may enhance clinical management strategies and improve patient outcomes.

## MATERIALS AND METHODS

### Microbial taxa mediation models and notations

Throughout this paper, we use *i* to index the *i*th cluster and *k* to index the *k*th observation within each cluster. Consider T as exposure variable, Y as the response variable, M as mediators, C as covariate. In repeated measurement data, the repeated measures form a cluster. Figure 1 illustrates the relationships within a high-dimensional mediation model with t mediators in repeated measures/longitudinal study, where mediators $M^{i}\in R^{m_{i}\times t}$ influence outcomes $Y_{i}\in R^{m_{i}}$, which are measured at k repeated time points. Before constructing mediation models, we applied a log-nom transformation to the mediators M, which is based on the log-proportion with an added pseudo-count of 0.5 (36, 37). For each observation k of cluster i, the log-normally transformed mediators are computed as


(1) $$M_{it}^{log}={log}_{10}\left(\frac{M_{it}\;}{\sum_{t=1}^{H}M_{it}}+0.5\right)$$

An outcome model for mediation analysis using a linear mixed-effects model can be specified as


(2) $$Y_{i}=\beta_{T}T^{i}+\sum_{t=1}^{H}\beta_{M_{t}}M_{it}^{log}+\beta_{C}C^{i}+\gamma_{i}Z^{i}+\epsilon_{i}$$

where the model can be decomposed into $\beta_{T}T^{i}+\sum_{t=1}^{H}\beta_{M_{t}}M_{it}^{log}+\beta_{C}C^{i}$ as fixed effects and $\gamma_{i}Z^{i}$ as random effects. For the estimation of fixed effects, we employed the quasi-likelihood approach proposed by Li et al. (13). This methodology was selected for its efficacy and simplicity in inferring unknown parameters within high-dimensional linear mixed-effects models. Furthermore, it offers ease of implementation and applicability to data sets that may include imbalance cluster sizes. For ease of presentation, the fixed effects of the outcome model are denoted as $\beta^{*}X^{i}$. Let i=1,...,n be the cluster indices. For the ith cluster, we have a response vector $Y_{i}\in R^{m_{i}}$, a design matrix for the fixed effects $X^{i}\in R^{m_{i}\times p}$, and a design matrix for the random effects $Z^{i}\in R^{m_{i}\times q}$, where $m_{i}$ is the size of the ith cluster. For simplification, the outcome model can also be written as


(3) $$Y_{i}=\beta^{*}X_{i}+\gamma_{i}Z^{i}+\epsilon_{i},i=1,...,n,$$

where $\beta^{*}\in R^{p}$ is the vector of fixed effects, $\gamma_{i}\in R^{q}$is the vector of random effects of the *i*th cluster, and $\epsilon_{i}\in R^{m_{i}}$ is the noise vector for the ith cluster. For i=1,...,n, we assume $\gamma_{i}$ and $\epsilon_{i}$ are independently distributed with mean zero and variance $\psi \in R^{q\times q}$ and $\sigma_{e}^{2}{I_{m}}_{i}$, respectively. We refer $\gamma_{i}$ and $\epsilon_{i}$ as the random components of the outcome model.

We assume that the vector of fixed effects $\beta^{*}$ is sparse such that ${\left|\left|\beta^{*}\right|\right|}_{0}\leq s$ with s unknown. We consider model 3 where p, s, and q can grow and p can be much larger than N, where $N=\sum_{i=1}^{n}m_{i}$ is the total sample size. The cluster sizes ${\left\{m_{i}\right\}}_{i=1}^{n}$ can be either fixed or grow with n. The primary challenge in estimating fixed effects in model 3 arises from the correlation among observations induced by random effects. For the ith cluster, the covariance of the random components is $\sum_{\theta *}^{i}=Z^{i}\Psi {\left(Z^{i}\right)}^{⊤}+\sigma_{e}^{2}I_{m_{i}}$, which involves unknown parameters Ψ and $\sigma_{e}^{2}$. To simplify the optimization problem and to separate the fixed effects estimation from the random effects estimation in the likelihood function for Gaussian mixed-effects models, a proxy of $\sum_{\theta *}^{i}$ is considered to be


(4) $$\sum_{a}^{i}=\;\alpha Z^{i}{\left(Z^{i}\right)}^{⊤}+I_{m_{i}}$$

where *a* > 0 is a tuning parameter controlling the contribution of random effects. The value of a is chosen by cross-validation using the error criteria ${\left\|Y-X\hat{\beta }(a)\right\|}_{2}^{2},$, where $\hat{\beta }\left(a\right)$ is the proposed estimate associated with a specific a. Specifically, let $X_{a}$ and $Y_{a}$ denote the transformed observations such that $\left(X_{a},Y_{a})=(\sum_{a}^{-1/2}X,\sum_{a}^{-1/2}Y\right)$.

First, we estimate the fixed effects via the lasso based on the transformed data. For some fixed *a* > 0, define


(5) $$\hat{\beta }={\mathrm{argmin}}_{\beta \in R^{p}}\left\{\frac{1}{2\mathrm{Tr}\left(\Sigma_{a}^{-1}\right)}{\left\|Y_{a}-X_{a}\beta \right\|}_{2}^{2}+\lambda ||\beta {\|}_{1}\right\}$$

where $\lambda \in \left\{2,...,0.1\right\}\times \sqrt{\frac{2log\left(p\right)}{N}}$ is a regularization parameter selected via cross-validation. The quantity $Tr(\sum_{a}^{-1})$ can be viewed as the effective sample size in the current problem. Given the task of making inference for $\beta_{j}^{*}$, the following de-biased estimator is used. For $\hat{\beta }$ defined in model 5,


(6) $${\hat{\beta }}^{\left(db\right)}={\hat{\beta }}_{j}+\frac{{\hat{w}}_{j}^{T}(Y_{a}-X_{a}\hat{\beta })}{{\hat{w}}_{j}^{T}(X_{a}).,j},$$

where ${\hat{w}}_{j}\in R^{N}$ can be viewed as a correction score, which can be computed through another lasso regression. For computational convenience, the lasso approach is considered based on the transformed data. Define the correction score ${\hat{w}}_{j}={\left(X_{a}\right)}_{.,j}-{\left(X_{a}\right)}_{.,-j}{\hat{\kappa }}_{j}$ , where


(7) $${\hat{\kappa }}_{j}={argmin}_{\kappa_{j}\in R^{p-1}}\left\{\frac{1}{2Tr\left(\sum_{a}^{-1}\right)}{\left|\left|{\left(X_{a}\right)}_{.,j}-{\left(X_{a}\right)}_{.,-j}\kappa_{j}\right|\right|}_{2}^{2}+\lambda_{j}{\left|\left|\kappa_{j}\right|\right|}_{1}\right\},$$

for tuning parameter $\lambda_{j}\in \left\{2,...,0.1\right\}\times \sqrt{\frac{2log\left(p\right)}{N}}$ is selected via cross-validation. The following empirical variance estimate is used for the estimator of the variance of ${\hat{\beta }}_{j}^{\left(db\right)}$


(8) $${\hat{V}}_{j}=\frac{\sum_{i=1}^{n}[({\hat{w}}_{j}^{i}{)}^{T}(Y_{a}^{i}-X_{a}^{i}\hat{\beta }){]}^{2}}{({\hat{w}}_{J}^{T}(X_{a}{)}_{.,j}{)}^{2}},$$

where $\hat{\beta }$ is the initial lasso estimator in model 5, ${\hat{w}}_{j}^{i}\in R_{i}^{m}$ is the *i*th sub-vector of ${\hat{w}}_{j}$ such that ${\hat{w}}_{j}=(({\hat{w}}_{j}^{1}{)}^{T},...,({\hat{w}}_{j}^{n}{)}^{T}{)}^{T}$, and $Y_{a}^{i}$ is the ith sub-vector of $Y_{a}$. Standard error of $\beta_{j}^{\left(db\right)}$ is calculated as


(9) $${\hat{SE}}_{j}=\sqrt{{\hat{V}}_{j}}.$$

The theoretical guarantee for the fixed effects estimates and inference has been explained in detail in Li et al. (13).

Restricted maximum likelihood (REML) estimation can produce unbiased estimators of the variance components in the low-dimensional setting (13). For this reason, REML is employed for parameter estimation within the linear mixed-effects models used for the mediator models. We fit each mediator to a linear mixed-effects model to estimate the effect of exposure *T^i^* on that mediator, accounting for covariates $C^{i}$ and the random effects structure $Z^{i}$, as follows


(10) $$M_{it}^{log}=\delta_{T_{it}}T^{i}+\delta_{C_{it}}C^{i}+\varphi_{it}Z^{i}+\eta_{it}$$

where $\delta_{T_{it}}$ and $\delta_{C_{it}}$ are vectors of fixed effects for the *t*th mediator in ith cluster, $\varphi_{it}$ is the vector of random effects for the tth mediator, and $\eta_{it}$ is the noise vector for the tth mediator in ith cluster.

Depending on the data and the model-fitting results, random effects can be modeled as a random intercept and slope (14). In this study, due to the large number of repeated measures per individual, random intercepts are used in the outcome and mediator models to avoid inflating variance.

The direct effect of T on Y, after accounting for the mediators M, is estimated as $\beta_{T}$, as determined by the outcome model. The indirect effect for mediator t is given by $\delta_{T_{it}}\times \beta_{M_{t}},\;t=1,...,H$. The total effect is obtained by summing the direct and indirect effects (14).

### Community-level mediation

Three non-phylogenetic alpha diversity indices; observed, Shannon, and Simpson were calculated at the genus level. Each index was used in a separate single-mediator model within a SEM framework to infer causality via the product-of-coefficient approach. For each index, we tested whether clinical states and disease aggressiveness alter sputum microbiome alpha diversity in CF patients, and whether altered diversity, in turn, affects lung function, adjusting for age.

Statistical inference was performed using a non-parametric bootstrap procedure with 3,000 resamples. To maintain the integrity of the intracluster correlation structure, resampling was performed with replacement at the cluster level. For each bootstrap sample, the entire mediation analysis, including the tuning parameter estimation, was repeated. Bootstrap estimates were then aggregated to derive point estimates, confidence intervals, and *P*-values. Confidence intervals were constructed using the BCa method. *P*-values were calculated as the proportion of bootstrap estimates less than or equal to zero, adjusted for two-tailed testing. Parallel processing was implemented to manage the 3,000 resamples and optimize computational efficiency.

The models assume linear relationships between variables, mediators measured without error, the dependent variable not causing the mediators, and no unmeasured confounding between the treatment and mediators or between the mediators and the outcome.

### Microbiome data processing

Reads obtained from the V3-V5 region of 16S rRNA gene, sequenced using the 454 GS FLX Titanium platform, were processed by stripping control sequences (TCAG) + barcode + forward primer. Reads with >1 expected error were discarded, and remaining reads were truncated to 350 bp using USEARCH (56). The reads were then dereplicated based on full-length via vsearch (v.2.22.1) (57). Sequences with ≥97% similarity were clustered into OTUs, and chimeras were removed using UPARSE (58). Taxonomy was assigned using SINTAX (59) at an 80% bootstrap threshold with the RDP v18 training set. Samples with <1,000 reads were removed due to low depth. OTUs were aggregated at the genus level. Alpha diversity was calculated from count data; beta diversity from Bray-Curtis dissimilarity based on proportions, with significance tested using PERMANOVA (9,999 permutations). Homogeneity of variance was calculated using betadisper. Estimated marginal means were used for statistical comparisons adjusted by Benjamini Hochberg correction using the emmeans package. Alpha and beta diversity were computed using phyloseq (60), and visualizations used ggpubr and ggplot2 (61). The STORM checklist is available at 10.5281/zenodo.15209818.

## Data Availability

Data set, including 16S rRNA gene amplicon sequence reads and associated metadata, is publicly available via the NCBI Sequence Read Archive under Bioproject number PRJNA423040. All analyses were conducted using R software and are fully reproducible using the codes available on the gitHub link https://github.com/sskoldas/Mediation.

## SUPPLEMENTAL MATERIAL

Supplemental material msystems.00196-25-s0001.pdf Figures S1 and S2 and Table S1.

ASM does not own the copyrights to Supplemental Material that may be linked to, or accessed through, an article. The authors have granted ASM a non-exclusive, world-wide license to publish the Supplemental Material files. Please contact the corresponding author directly for reuse.
