# Supplementary material for: Exploring the role of microbiome in cystic fibrosis clinical outcomes through a mediation analysis
Source: mSystems. 2025 May 28;10(6):e00196-25. doi: 10.1128/msystems.00196-25 (PMC12172493; doi:10.1128/msystems.00196-25)
Supplement: Supplemental material — Figures S1 and S2 and Table S1. [file msystems.00196-25-s0001.pdf]

S1A

Baseline-Exacerbation

Mediators	Estimate	P-value	CI [Lower-Upper]
Abiotrophia	-0.057	0.556	[-0.351, 0.060]
Acinetobacter	-0.03	0.661	[-0.194, 0.733]
Actinomyces	0.15	0.266	[-0.126, 0.513]
Alloprevotella	0.028	0.753	[-0.084, 0.250]
Atopobium	-0.044	0.691	[-0.395, 0.150]
Bergeyella	-0.068	0.531	[-0.380, 0.117]
Burkholderia	-0.412	0.21	[-1.238, 0.131]
Campylobacter	-0.084	0.209	[-0.271, 0.163]
Capnocytophaga	-0.022	0.907	[-0.475, 0.345]
Catonella	-0.093	0.749	[-0.780, 0.380]
Cloacibacterium	0.096	0.455	[-1.251, 0.256]
Clostridia_UCG_014	0.078	0.476	[-0.326, 0.330]
Corynebacterium	-0.076	0.654	[-0.512, 0.282]
Dialister	-0.012	0.878	[-0.264, 0.117]
Eubacterium_brachy_group	0.017	0.879	[-0.122, 0.178]
Eubacterium_nodatum_group	-0.003	0.956	[-0.311, 0.260]
F0058	0.133	0.409	[-0.139, 0.695]
Fusobacterium	0.045	0.589	[-0.184, 0.256]
Gemella	0.185	0.188	[-0.082, 0.609]
Haemophilus	0.05	0.751	[-0.165, 0.453]
Kingella	0.143	0.38	[-0.083, 0.584]
Lachnoanaerobaculum	-0.224	0.193	[-0.783, 0.069]
Lautropia	-0.047	0.773	[-0.419, 0.139]
Leptotrichia	0.011	0.945	[-0.124, 0.254]
Limosilactobacillus	0.001	0.967	[-0.263, 0.082]
Megasphaera	0.015	0.919	[-0.279, 0.489]
Mogibacterium	-0.042	0.757	[-0.371, 0.171]
Mycoplasma	-0.118	0.417	[-0.521, 0.157]
Neisseria	0.019	0.885	[-0.122, 0.210]
Oribacterium	0.004	0.954	[-0.177, 0.271]
Parvimonas	-0.055	0.694	[-0.409, 0.127]
Peptostreptococcus	0.123	0.571	[-0.234, 0.695]
Porphyromonas	0.037	0.993	[-0.333, 0.653]
Prevotella	0.002	0.915	[-0.277, 0.300]
Prevotella_7	-0.158	0.373	[-0.656, 0.138]
Pseudomonas	0.082	0.654	[-0.235, 0.747]
Rothia	0.005	0.963	[-0.126, 0.219]
Scardovia	0.018	0.996	[-0.186, 0.289]
Selenomonas	-0.08	0.643	[-0.573, 0.197]
Shuttleworthia	-0.014	0.923	[-0.263, 0.157]
Staphylococcus	-0.055	0.728	[-0.433, 0.128]
Stenotrophomonas	0.074	0.77	[-0.248, 0.598]
Stomatobaculum	0.07	0.58	[-0.254, 0.526]
Streptococcus	-0.071	0.724	[-0.650, 0.293]
Tannerella	0.103	0.725	[-0.525, 0.918]
Treponema	0.166	0.344	[-0.219, 0.651]
Veillonella	-0.135	0.393	[-0.594, 0.151]

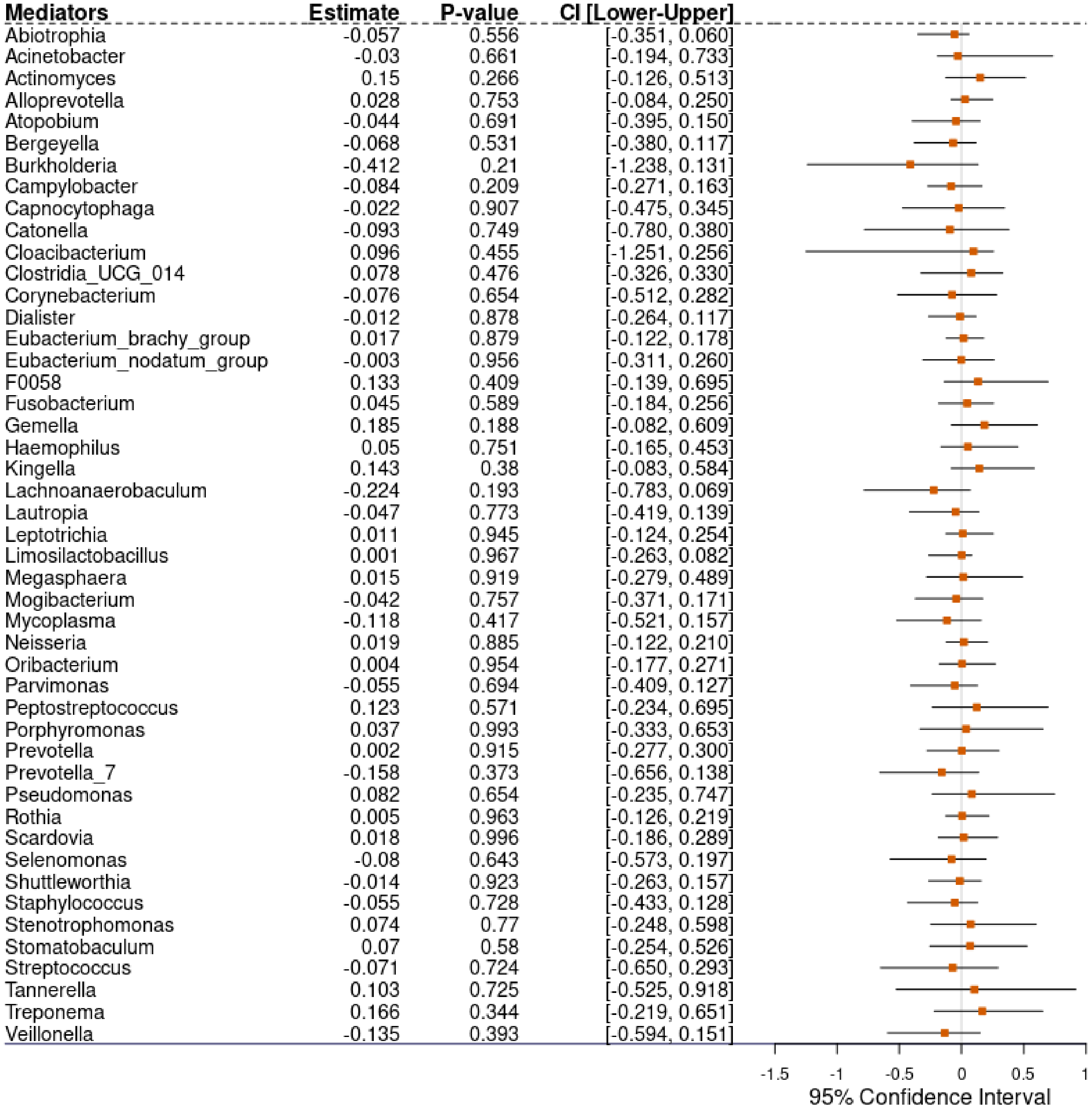

S1B

Baseline-Treatment

Mediators	Estimate	P-value	CI [Lower-Upper]
Abiotrophia	0.018	0.817	[-0.119, 0.290]
Acinetobacter	0.017	0.871	[-0.477, 0.294]
Actinomyces	0.091	0.673	[-0.363, 0.668]
Alloprevotella	0.071	0.655	[-0.046, 0.518]
Atopobium	0.048	0.814	[-0.213, 0.506]
Bergeyella	-0.191	0.486	[-0.833, 0.346]
Burkholderia	-0.183	0.185	[-0.623, 0.054]
Campylobacter	-0.106	0.21	[-0.387, 0.186]
Capnocytophaga	0.085	0.804	[-0.444, 0.857]
Catonella	-0.031	0.937	[-0.636, 0.407]
Cloacibacterium	0.019	0.841	[-0.146, 1.401]
Clostridia_UCG_014	-0.2	0.503	[-0.894, 0.202]
Corynebacterium	-0.153	0.503	[-0.776, 0.391]
Dialister	0.029	0.707	[-0.186, 0.367]
Eubacterium_brachy_group	-0.049	0.543	[-0.328, 0.117]
Eubacterium_nodatum_group	-0.125	0.423	[-0.491, 0.234]
F0058	0.168	0.554	[-0.187, 0.783]
Fusobacterium	-0.038	0.987	[-0.448, 0.238]
Gemella	0.267	0.325	[-0.297, 0.870]
Haemophilus	-0.055	0.808	[-0.614, 0.175]
Kingella	0.092	0.323	[-0.269, 0.497]
Lachnoanaerobaculum	-0.047	0.797	[-0.556, 0.134]
Lautropia	0.078	0.703	[-0.464, 0.480]
Leptotrichia	-0.019	0.891	[-0.295, 0.168]
Limosilactobacillus	-0.482	0.436	[-1.829, 0.634]
Megasphaera	-0.084	0.798	[-0.707, 0.322]
Mogibacterium	0.119	0.342	[-0.124, 0.517]
Mycoplasma	-0.016	0.816	[-0.333, 0.099]
Neisseria	0.012	0.855	[-0.407, 0.396]
Oribacterium	0.052	0.759	[-0.159, 0.450]
Parvimonas	-0.013	0.896	[-0.468, 0.253]
Peptostreptococcus	-0.024	0.839	[-0.336, 0.158]
Porphyromonas	0.338	0.137	[-0.101, 1.115]
Prevotella	-0.217	0.439	[-0.846, 0.209]
Prevotella_7	0.125	0.61	[-0.360, 0.821]
Pseudomonas	-0.222	0.444	[-1.001, 0.254]
Rothia	0.015	0.899	[-0.135, 0.243]
Scardovia	-0.028	0.76	[-0.664, 0.626]
Selenomonas	-0.045	0.765	[-0.413, 0.161]
Shuttleworthia	-0.063	0.504	[-0.351, 0.077]
Staphylococcus	0.031	0.865	[-0.387, 0.640]
Stenotrophomonas	-0.03	0.826	[-0.615, 0.546]
Stomatobaculum	-0.029	0.835	[-0.535, 0.246]
Streptococcus	-0.118	0.727	[-0.916, 0.592]
Tannerella	-0.346	0.26	[-1.204, 0.273]
Treponema	-0.039	0.655	[-0.244, 0.133]
Veillonella	-0.028	0.9	[-0.536, 0.318]

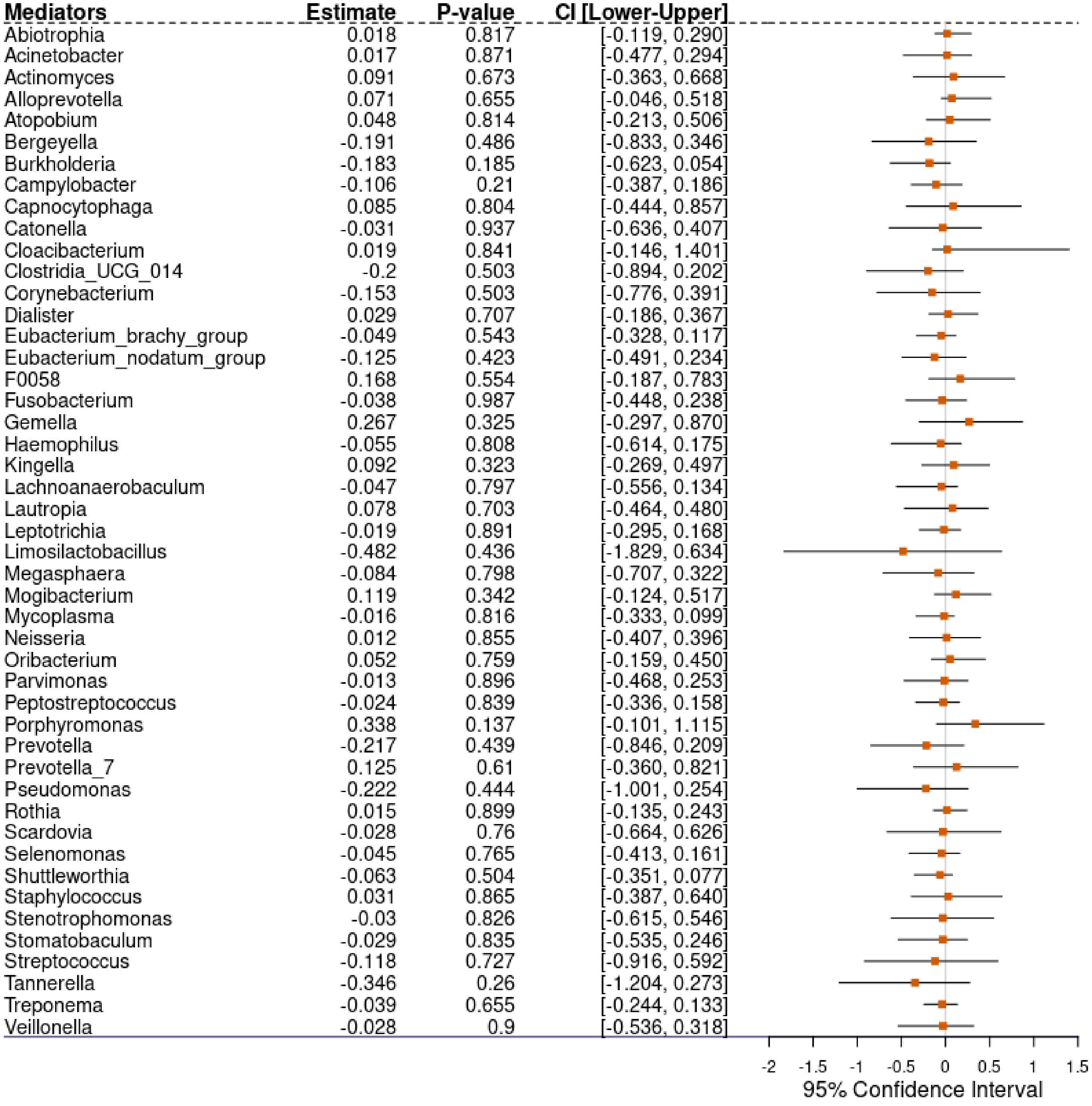

S1C

Baseline-Recovery

Mediators	Estimate	P-value	CI [Lower-Upper]
Abiotrophia	-0.113	0.347	[-0.562, 0.089]
Acinetobacter	0.032	0.877	[-0.357, 0.512]
Actinomyces	-0.069	0.813	[-0.736, 0.324]
Alloprevotella	0.158	0.334	[-0.117, 0.612]
Atopobium	0.004	0.979	[-0.382, 0.660]
Bergeyella	-0.058	0.819	[-0.627, 0.440]
Burkholderia	-0.338	0.39	[-1.215, 0.331]
Campylobacter	-0.064	0.465	[-0.343, 0.289]
Capnocytophaga	0.006	0.954	[-0.731, 0.878]
Catonella	-0.232	0.417	[-1.005, 0.337]
Cloacibacterium	0.008	0.931	[-0.900, 0.261]
Clostridia_UCG_014	-0.312	0.211	[-0.882, 0.161]
Corynebacterium	-0.162	0.543	[-0.618, 0.153]
Dialister	0.152	0.387	[-0.123, 0.650]
Eubacterium_brachy_group	0.047	0.529	[-0.102, 0.300]
Eubacterium_nodatum_group	-0.102	0.751	[-0.549, 0.476]
F0058	0.122	0.531	[-0.152, 0.557]
Fusobacterium	-0.2	0.155	[-0.685, 0.051]
Gemella	-0.172	0.283	[-0.677, 0.098]
Haemophilus	-0.068	0.709	[-0.492, 0.174]
Kingella	0.149	0.217	[-0.040, 0.558]
Lachnoanaerobaculum	-0.175	0.618	[-1.116, 0.301]
Lautropia	0.025	0.823	[-0.124, 0.453]
Leptotrichia	-0.018	0.913	[-0.590, 0.310]
Limosilactobacillus	0.001	0.983	[-0.209, 0.268]
Megasphaera	0.083	0.669	[-0.302, 0.665]
Mogibacterium	-0.05	0.841	[-0.612, 0.305]
Mycoplasma	-0.045	0.653	[-0.310, 0.143]
Neisseria	-0.007	0.958	[-0.258, 0.076]
Oribacterium	0.434	0.225	[-0.115, 1.478]
Parvimonas	0.053	0.704	[-0.220, 0.362]
Peptostreptococcus	-0.056	0.821	[-0.685, 0.209]
Porphyromonas	0.329	0.161	[-0.146, 1.016]
Prevotella	-0.323	0.534	[-1.302, 0.632]
Prevotella_7	0.018	0.912	[-0.470, 0.583]
Pseudomonas	-0.043	0.985	[-0.883, 0.446]
Rothia	-0.009	0.994	[-0.367, 0.299]
Scardovia	-0.003	0.73	[-0.175, 0.281]
Selenomonas	0.012	0.96	[-0.234, 0.373]
Shuttleworthia	-0.141	0.186	[-0.639, 0.044]
Staphylococcus	-0.129	0.681	[-0.858, 0.309]
Stenotrophomonas	0.073	0.684	[-0.244, 0.531]
Stomatobaculum	0.01	0.983	[-0.308, 0.512]
Streptococcus	-0.021	0.922	[-0.714, 0.636]
Tannerella	-0.26	0.237	[-0.943, 0.235]
Treponema	-0.192	0.103	[-0.480, 0.041]
Veillonella	-0.433	0.314	[-1.546, 0.497]

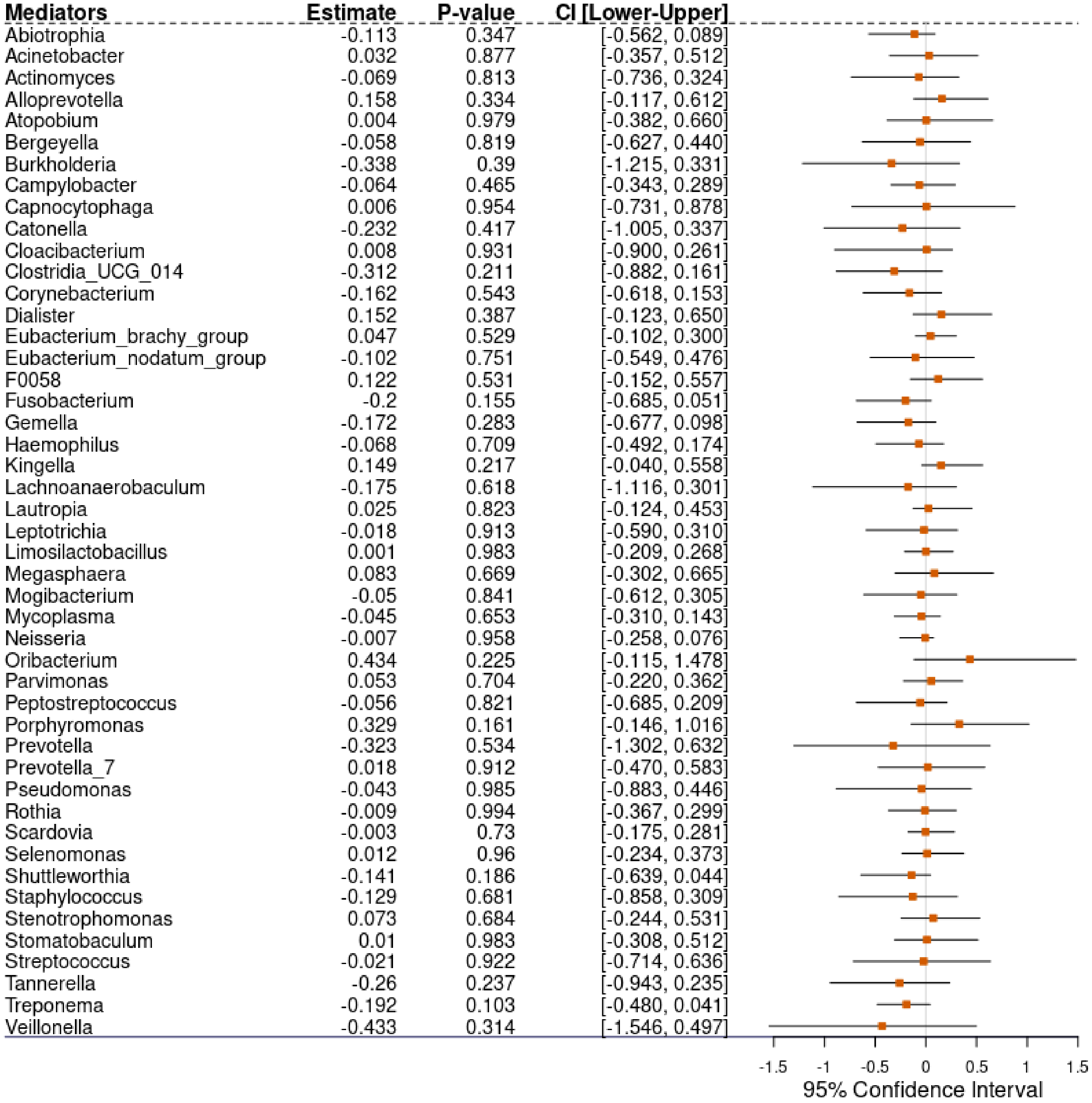

S1D

Exacerbation-Recovery

Mediators	Estimate	P-value	CI [Lower-Upper]
Abiotrophia	-0.112	0.499	[-0.680, 0.472]
Acinetobacter	0.09	0.8	[-0.771, 0.780]
Actinomyces	-0.017	0.929	[-0.962, 0.356]
Alloprevotella	-0.278	0.479	[-1.300, 0.540]
Atopobium	-0.148	0.786	[-1.391, 0.847]
Bergeyella	-0.173	0.616	[-1.056, 0.492]
Burkholderia	-0.09	0.713	[-0.736, 0.370]
Campylobacter	-0.121	0.825	[-1.093, 0.480]
Capnocytophaga	0.219	0.485	[-0.501, 1.089]
Catonella	0.165	0.825	[-1.020, 1.697]
Cloacibacterium	-0.178	0.609	[-0.911, 0.716]
Clostridia_UCG_014	-0.165	0.997	[-2.118, 0.405]
Corynebacterium	-0.252	0.729	[-1.482, 0.585]
Dialister	0.089	0.517	[-0.155, 0.463]
Eubacterium_brachy_group	-0.17	0.322	[-0.755, 0.127]
Eubacterium_nodatum_group	0.016	0.988	[-0.711, 0.660]
F0058	-0.231	0.494	[-1.309, 0.339]
Fusobacterium	-0.227	0.392	[-0.948, 0.581]
Gemella	-0.94	0.049	[-2.166, 0.013]
Haemophilus	-0.153	0.479	[-0.790, 0.192]
Kingella	0.08	0.632	[-0.137, 0.664]
Lachnoanaerobaculum	0.019	0.813	[-0.556, 1.164]
Lautropia	0.032	0.911	[-0.147, 0.510]
Leptotrichia	-0.122	0.538	[-0.689, 0.289]
Limosilactobacillus	0.002	0.819	[-0.377, 0.685]
Megasphaera	0.049	0.837	[-0.515, 0.822]
Mogibacterium	-0.111	0.732	[-0.942, 0.613]
Mycoplasma	0.672	0.168	[-0.157, 2.140]
Neisseria	-0.17	0.571	[-1.162, 0.133]
Oribacterium	-0.682	0.299	[-2.470, 0.565]
Parvimonas	0.102	0.79	[-0.609, 0.972]
Peptostreptococcus	-0.272	0.523	[-1.417, 0.517]
Porphyromonas	0.418	0.265	[-0.309, 1.489]
Prevotella	0.006	0.925	[-0.921, 0.796]
Prevotella_7	-0.046	0.783	[-0.669, 0.317]
Pseudomonas	-0.11	0.815	[-1.089, 0.748]
Rothia	-0.094	0.671	[-0.665, 0.211]
Scardovia	-0.07	0.445	[-0.863, 0.040]
Selenomonas	0.104	0.597	[-0.672, 0.825]
Shuttleworthia	-0.063	0.623	[-1.099, 0.124]
Staphylococcus	-0.086	0.84	[-0.874, 0.327]
Stenotrophomonas	-0.031	0.743	[-0.422, 0.202]
Stomatobaculum	0.191	0.507	[-0.212, 1.045]
Streptococcus	-0.356	0.386	[-1.404, 0.377]
Tannerella	-0.185	0.694	[-1.588, 1.084]
Treponema	0.061	0.935	[-0.827, 1.199]
Veillonella	-0.878	0.13	[-2.498, 0.236]

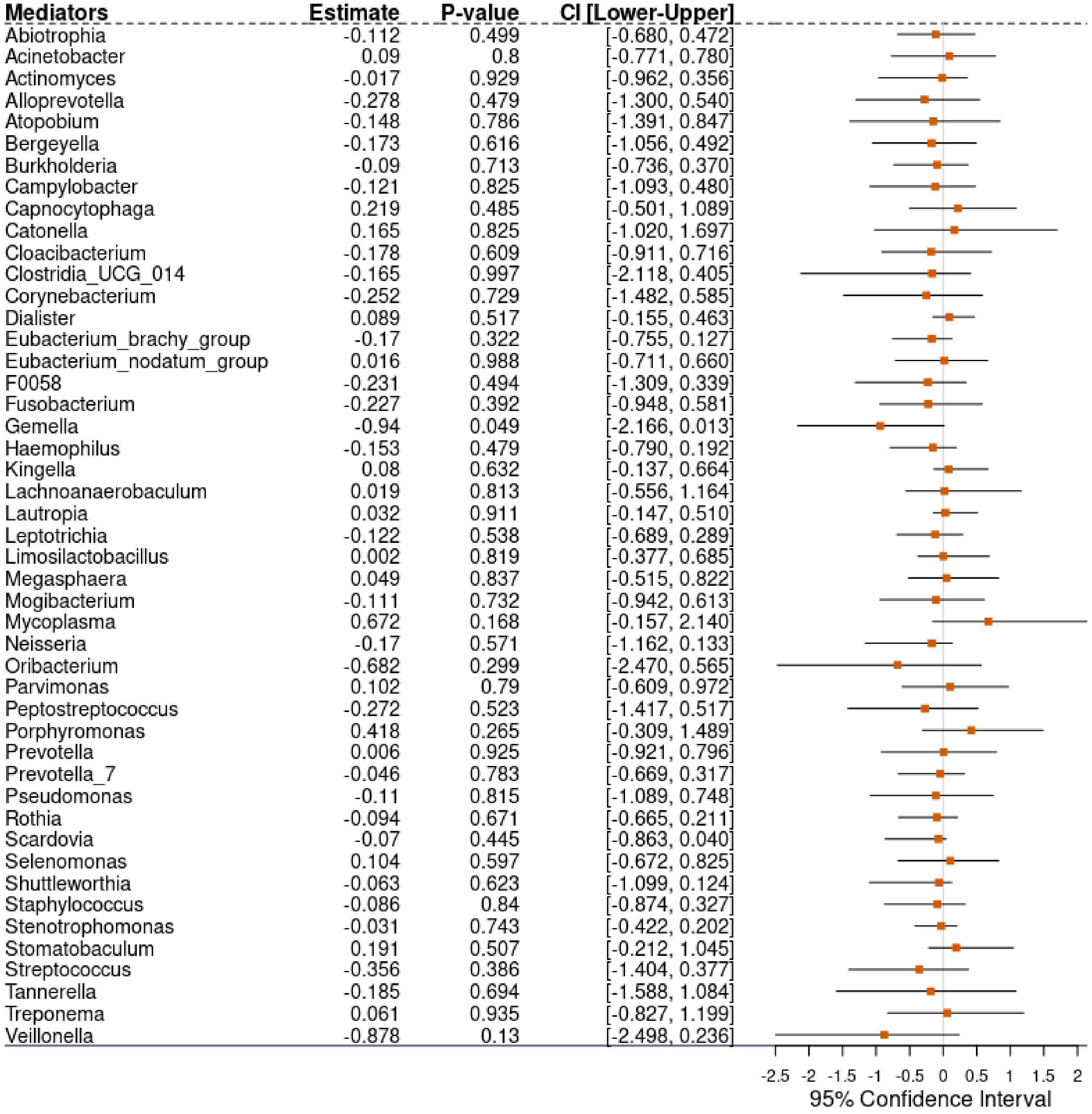

S1E

Treatment-Recovery

Mediators	Estimate	P-value	CI [Lower-Upper]
Abiotrophia	-0.168	0.384	[-0.846, 0.110]
Acinetobacter	0.073	0.869	[-0.715, 1.302]
Actinomyces	-0.015	0.981	[-1.264, 0.766]
Alloprevotella	-0.095	0.562	[-0.459, 1.264]
Atopobium	-0.185	0.7	[-1.305, 0.379]
Bergeyella	-0.091	0.692	[-0.697, 0.320]
Burkholderia	0.068	0.658	[-0.140, 0.483]
Campylobacter	-0.111	0.656	[-0.713, 0.407]
Capnocytophaga	-0.111	0.734	[-0.897, 0.451]
Catonella	0.031	0.965	[-0.502, 0.797]
Cloacibacterium	-0.181	0.678	[-1.326, 0.569]
Clostridia_UCG_014	-0.04	0.915	[-1.842, 0.466]
Corynebacterium	-0.458	0.36	[-1.733, 0.437]
Dialister	-0.009	0.904	[-0.597, 0.083]
Eubacterium_brachy_group	0.247	0.54	[-0.699, 1.165]
Eubacterium_nodatum_group	-0.109	0.827	[-0.907, 0.475]
F0058	0.171	0.622	[-0.466, 1.109]
Fusobacterium	0.111	0.734	[-0.588, 0.837]
Gemella	-0.616	0.038	[-1.580, 0.033]
Haemophilus	-0.129	0.546	[-0.855, 0.102]
Kingella	0.518	0.179	[-0.514, 1.447]
Lachnoanaerobaculum	-0.053	0.816	[-0.382, 1.395]
Lautropia	-0.083	0.345	[-0.315, 0.734]
Leptotrichia	0.145	0.583	[-0.312, 0.885]
Limosilactobacillus	0.149	0.765	[-0.661, 1.376]
Megasphaera	0.036	0.861	[-0.983, 0.211]
Mogibacterium	0.02	0.915	[-0.864, 0.810]
Mycoplasma	0.033	0.885	[-0.465, 1.547]
Neisseria	-0.158	0.41	[-0.818, 0.496]
Oribacterium	-0.058	0.662	[-0.320, 0.759]
Parvimonas	-0.308	0.562	[-1.668, 0.821]
Peptostreptococcus	-0.108	0.834	[-1.281, 0.340]
Porphyromonas	-0.073	0.738	[-0.603, 0.261]
Prevotella	0.191	0.436	[-0.298, 0.937]
Prevotella_7	-0.031	0.929	[-0.753, 0.433]
Pseudomonas	-0.027	0.913	[-0.591, 0.311]
Rothia	-0.058	0.584	[-0.647, 0.152]
Scardovia	-0.099	0.427	[-0.624, 1.067]
Selenomonas	0.083	0.635	[-0.236, 0.642]
Shuttleworthia	-0.016	0.936	[-0.417, 0.776]
Staphylococcus	0	0.999	[-0.537, 0.404]
Stenotrophomonas	-0.091	0.715	[-0.666, 0.163]
Stomatobaculum	0.007	0.928	[-0.383, 0.343]
Streptococcus	0.424	0.396	[-0.579, 1.591]
Tannerella	-0.058	0.853	[-0.924, 0.440]
Treponema	0.167	0.588	[-0.301, 1.129]
Veillonella	0.101	0.827	[-0.506, 1.099]

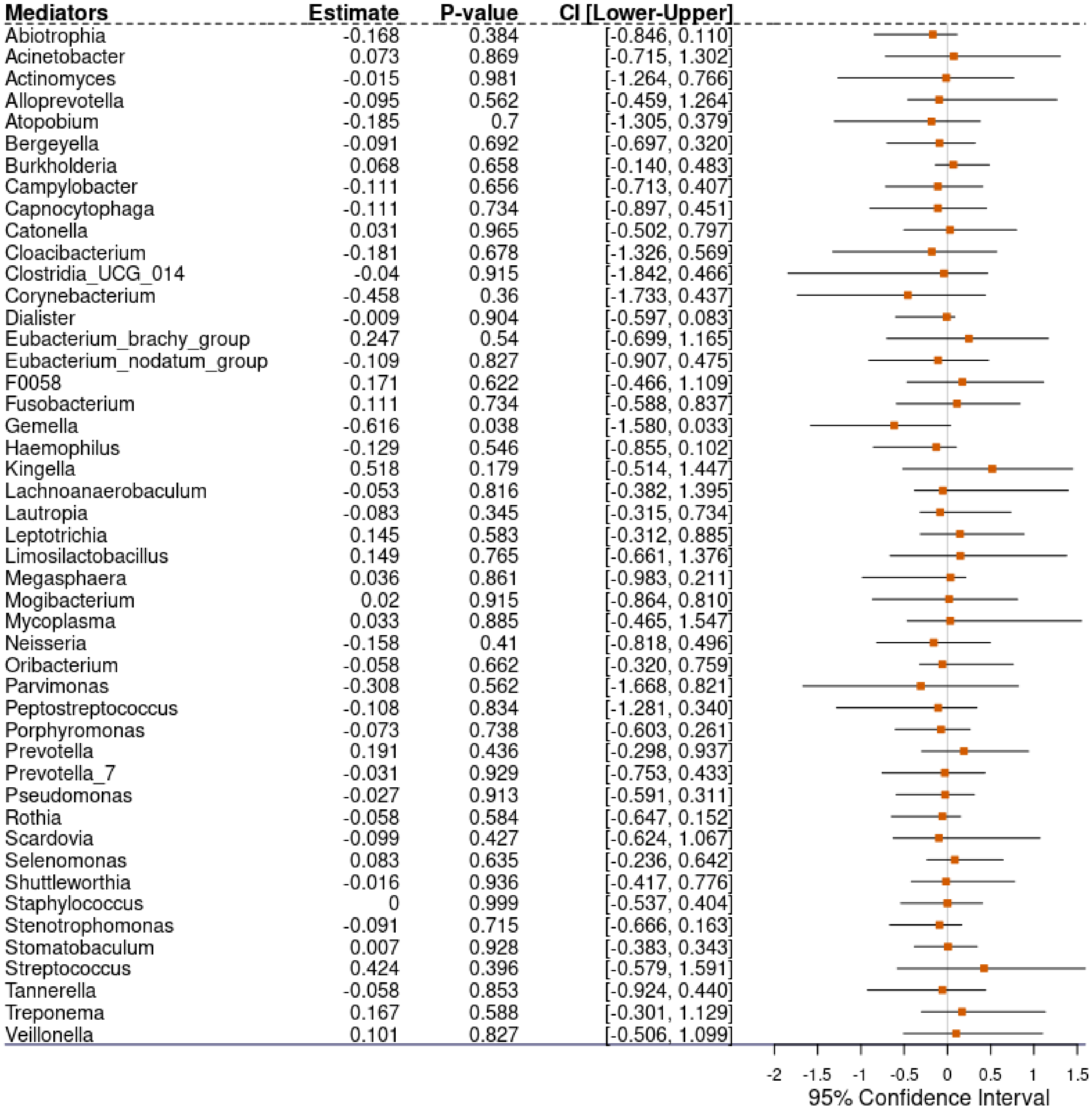

S1F

Mild-Moderate/Severe

Mediators	Estimate	P-value	CI [Lower-Upper]
Abiotrophia	0.279	0.362	[-0.183, 0.862]
Acinetobacter	0.03	0.901	[-0.247, 0.907]
Actinomyces	-0.085	0.744	[-0.802, 0.171]
Alloprevotella	-0.055	0.559	[-0.321, 0.182]
Atopobium	-0.045	0.804	[-0.496, 0.342]
Bergeyella	-0.402	0.377	[-1.593, 0.671]
Burkholderia	-0.096	0.836	[-1.696, 0.537]
Campylobacter	-0.081	0.515	[-0.453, 0.214]
Capnocytophaga	-0.082	0.653	[-0.441, 0.308]
Catonella	0.169	0.372	[-0.178, 0.821]
Cloacibacterium	0.061	0.769	[-0.461, 0.659]
Clostridia_UCG_014	0.011	0.621	[-0.297, 0.913]
Corynebacterium	-0.384	0.167	[-1.117, 0.212]
Dialister	0.058	0.741	[-0.267, 0.925]
Eubacterium_brachy_group	0.007	0.877	[-0.315, 0.407]
Eubacterium_nodatum_group	-0.086	0.892	[-0.857, 1.021]
F0058	-0.051	0.713	[-0.449, 0.159]
Fusobacterium	-0.159	0.481	[-0.917, 0.377]
Gemella	-0.061	0.72	[-0.577, 0.421]
Haemophilus	-0.133	0.397	[-0.428, 2.463]
Kingella	-0.018	0.864	[-0.216, 0.134]
Lachnoanaerobaculum	0.129	0.461	[-0.194, 0.654]
Lautropia	0.158	0.575	[-0.374, 0.887]
Leptotrichia	-0.017	0.807	[-0.273, 0.140]
Limosilactobacillus	0.11	0.202	[-0.073, 0.383]
Megasphaera	-0.14	0.71	[-1.087, 0.393]
Mogibacterium	0.139	0.685	[-0.404, 1.098]
Mycoplasma	0.089	0.569	[-0.257, 0.690]
Neisseria	0.071	0.817	[-0.142, 0.605]
Oribacterium	-0.024	0.803	[-0.310, 0.259]
Parvimonas	-0.025	0.947	[-0.659, 0.389]
Peptostreptococcus	-0.179	0.433	[-0.820, 0.260]
Porphyromonas	0.071	0.652	[-0.210, 0.516]
Prevotella	-0.016	0.951	[-0.513, 0.419]
Prevotella_7	-0.015	0.971	[-0.404, 0.419]
Pseudomonas	-0.079	0.933	[-1.553, 0.838]
Rothia	0.17	0.693	[-0.264, 1.870]
Scardovia	-0.069	0.313	[-0.515, 0.058]
Selenomonas	0.082	0.649	[-0.167, 0.577]
Shuttleworthia	-0.022	0.785	[-0.634, 0.262]
Staphylococcus	-0.228	0.586	[-1.479, 0.336]
Stenotrophomonas	0.268	0.679	[-0.937, 1.912]
Stomatobaculum	0.021	0.963	[-0.933, 0.179]
Streptococcus	-0.19	0.73	[-1.714, 0.898]
Tannerella	-0.092	0.832	[-3.454, 0.067]
Treponema	-0.013	0.975	[-0.671, 0.596]
Veillonella	0.225	0.554	[-0.785, 1.495]

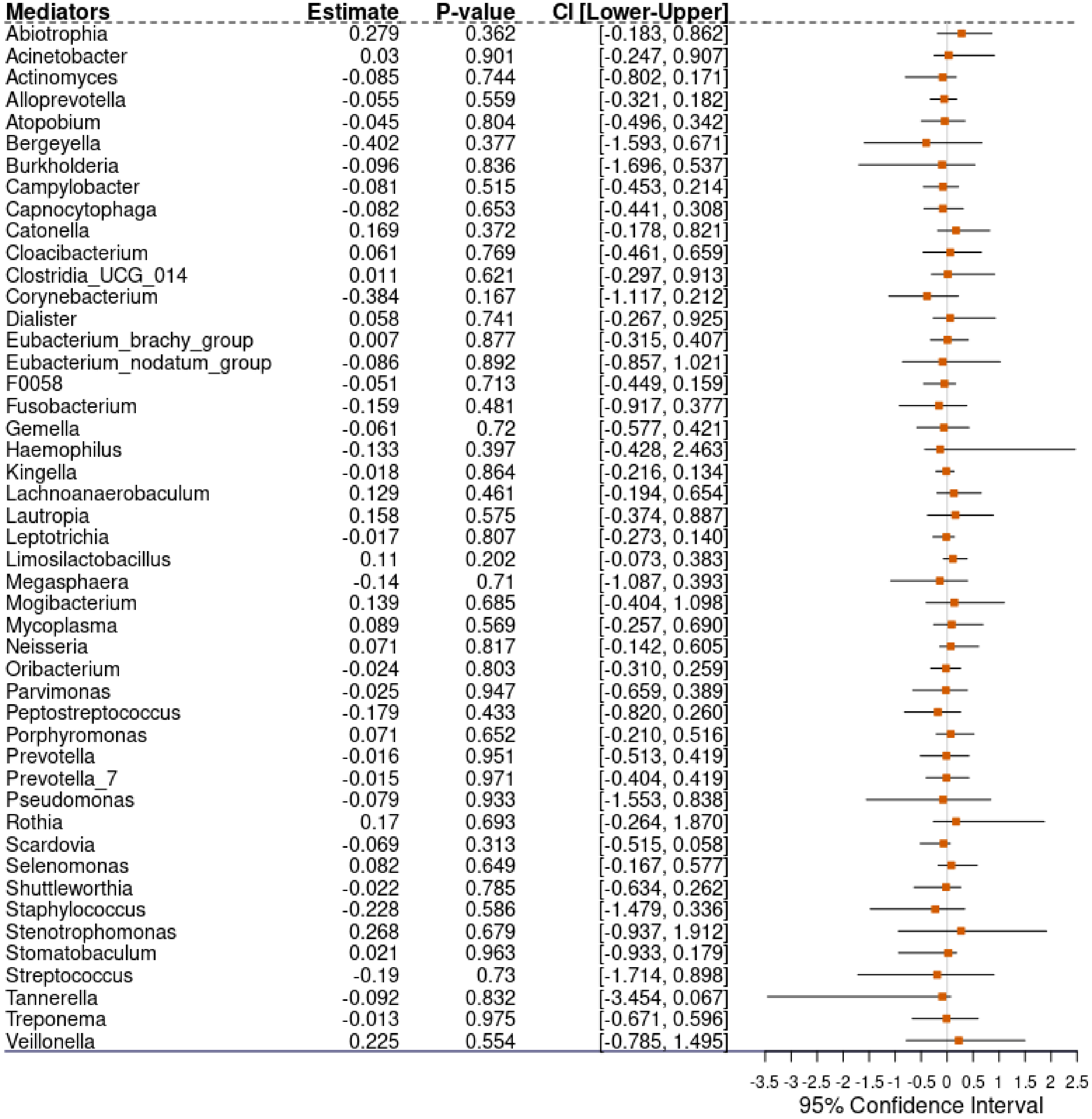

Supplementary Figures 1. Indirect Effects of Microbiome from Pairwise Comparisons of Clinical States and Disease Aggressiveness. Plots **S1A-E** show causal inference results using microbial taxa as mediators in pairwise comparisons of clinical states. Plot **S1F** shows results for disease aggressiveness. We investigated whether clinical states and disease aggressiveness affect sputum microbiome microbial taxa in cystic fibrosis patients, and whether altered microbial taxa influence lung function, adjusting for age. Empirical confidence intervals and p-values were obtained using a nonparametric bootstrap with 3000 resamples. "Estimate" represents the mean of all bootstrap resamples and denotes the mediation effect. Results are presented with 95% confidence intervals.

S2A

Baseline-Exacerbation

Observed Genera

Effects	Estimate	p-value
Total Effect	-5.244	<0.001
Direct Effect	-5.498	<0.001
Indirect Effect	0.254	0.217

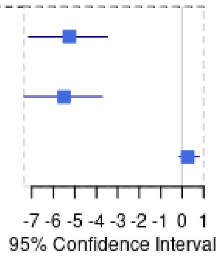

Shannon Index

Effects	Estimate	p-value
Total Effect	-5.252	<0.001
Direct Effect	-5.374	<0.001
Indirect Effect	0.122	0.593

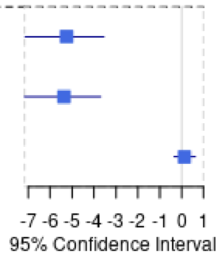

Simpson Index

Effects	Estimate	p-value
Total Effect	-5.261	<0.001
Direct Effect	-5.391	<0.001
Indirect Effect	0.13	0.503

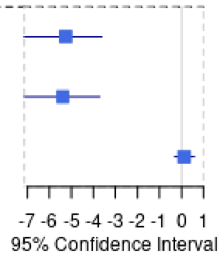

S2B

Baseline-Treatment

Observed Genera

Effects	Estimate	p-value
Total Effect	-5.253	<0.001
Direct Effect	-4.017	0.003
Indirect Effect	-1.236	0.003

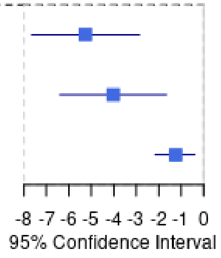

Shannon Index

Effects	Estimate	p-value
Total Effect	-5.218	<0.001
Direct Effect	-4.158	<0.001
Indirect Effect	-1.06	0.001

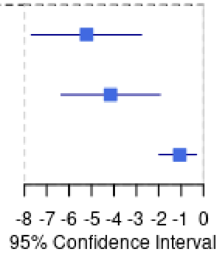

Simpson Index

Effects	Estimate	p-value
Total Effect	-5.145	<0.001
Direct Effect	-4.231	<0.001
Indirect Effect	-0.914	<0.001

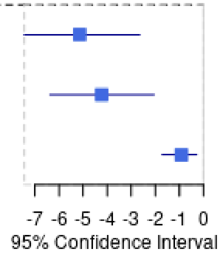

S2C

Baseline-Recovery

Observed Genera

Effects	Estimate	p-value
Total Effect	-4.719	<0.001
Direct Effect	-3.201	0.009
Indirect Effect	-1.518	0.001

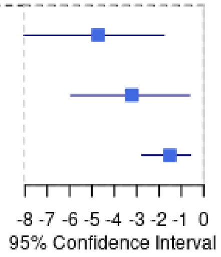

Shannon Index

Effects	Estimate	p-value
Total Effect	-4.667	0.001
Direct Effect	-3.703	0.005
Indirect Effect	-0.964	0.002

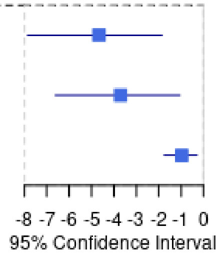

Simpson Index

Effects	Estimate	p-value
Total Effect	-4.601	<0.001
Direct Effect	-3.747	0.002
Indirect Effect	-0.855	0.001

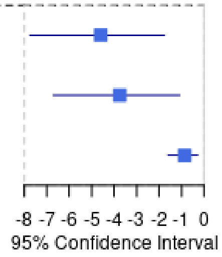

S2D

Exacerbation-Recovery

Observed Genera

Effects	Estimate	p-value
Total Effect	-2.978	0.137
Direct Effect	0.071	0.921
Indirect Effect	-3.049	<0.001

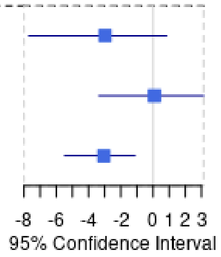

Shannon Index

Effects	Estimate	p-value
Total Effect	-2.675	0.173
Direct Effect	-0.535	0.787
Indirect Effect	-2.141	<0.001

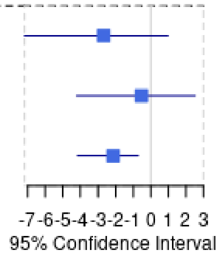

Simpson Index

Effects	Estimate	p-value
Total Effect	-2.607	0.181
Direct Effect	-0.841	0.678
Indirect Effect	-1.765	0.001

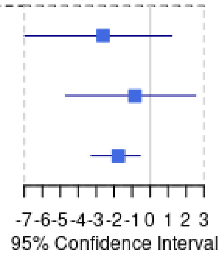

S2E

Treatment-Recovery

Observed Genera

Effects	Estimate	p-value
Total Effect	-0.993	0.534
Direct Effect	-1.146	0.484
Indirect Effect	0.152	0.455

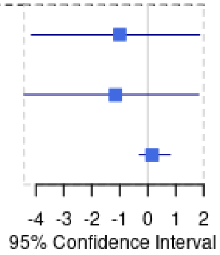

Shannon Index

Effects	Estimate	p-value
Total Effect	-1.064	0.487
Direct Effect	-1.37	0.347
Indirect Effect	0.306	0.235

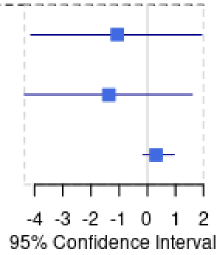

Simpson Index

Effects	Estimate	p-value
Total Effect	-1.001	0.511
Direct Effect	-1.325	0.371
Indirect Effect	0.324	0.245

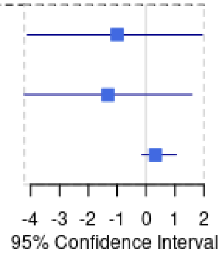

S2F

Mild-Moderate/Severe

Observed Genera

Effects	Estimate	p-value
Total Effect	-41.737	<0.001
Direct Effect	-39.333	<0.001
Indirect Effect	-2.404	0.003

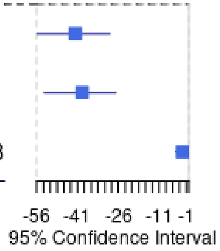

Shannon Index

Effects	Estimate	p-value
Total Effect	-41.735	<0.001
Direct Effect	-40.839	<0.001
Indirect Effect	-0.896	0.181

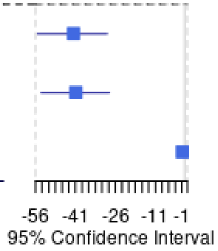

Simpson Index

Effects	Estimate	p-value
Total Effect	-41.334	<0.001
Direct Effect	-40.709	<0.001
Indirect Effect	-0.624	0.359

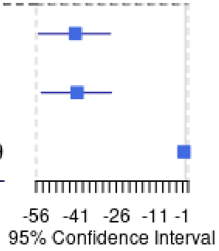

Supplementary Figures 2. Causal Inferences of Microbiome Alpha Diversities from Pairwise Comparisons of Clinical States and Disease Aggressiveness.

Plots **S2A-E** show causal inference results using alpha diversity indices as mediators in pairwise comparisons of clinical states. Plot **S2F** shows results for disease aggressiveness. We investigated whether clinical states and disease aggressiveness affect sputum microbiome alpha diversity in CF patients, and whether altered alpha diversity, in turn, influences lung function, adjusting for age. Empirical confidence intervals and p-values were obtained using a nonparametric bootstrap with 3000 resamples. "Estimate" represents the mean of all bootstrap resamples. Results are presented with 95% confidence intervals.

Supplementary Table S1. Patient and Sample Characteristics for Disease Aggressiveness Phenotypes

	**Mild**	**Moderate/Severe**
No. of patients	14	10
No. of samples	178	172
No. of samples per patient, mean (range)	12 (10-20)	17 (11-24)
Patient age, mean (range)	33 (20-54)	22 (11-31)
Patient FEV1%, mean (range)	68 (28-101)	44 (18-80)
Clinical state, count (%)		
Baseline	101 (57)	54 (32)
Exacerbation	30 (17)	33 (19)
Treatment	20 (11)	50 (29)
Recovery	27 (15)	35 (20)
**Footnote:** The values presented in this table are the raw observed values for each disease aggressiveness phenotype. Mean values do not reflect the model-adjusted estimates reported in the main text, which account for within-subject correlations.		
